# Chain end-group selectivity using an organometallic Al(iii)/K(i) ring-opening copolymerization catalyst delivers high molar mass, monodisperse polyesters†

**DOI:** 10.1039/d2sc02752f

**Published:** 2022-07-13

**Authors:** Wilfred T. Diment, Charlotte K. Williams

**Affiliations:** Department of Chemistry, University of Oxford, Chemistry Research Laboratory 12 Mansfield Road Oxford OX1 3TA UK charlotte.williams@chem.ox.ac.uk

## Abstract

Polyesters are important plastics, elastomers and fibres; efficient and selective polymerizations making predictable, high molar mass polymers are required. Here, a new type of catalyst for the ring-opening polymerization (ROCOP) of epoxides and anhydrides combines unusually high chain end-group selectivity, fast rates, and good molar mass control. The organometallic heterodinuclear Al(iii)/K(i) complex, applied with a diol, is tolerant to a range of epoxides/phthalic anhydride and produces only α,ω-hydroxyl telechelic polyesters with molar masses from 6–91 kg mol^−1^, in all cases with monomodal distributions. As proof of its potential, high molar mass poly(vinyl cyclohexene oxide-*alt*-phthalic anhydride) (91 kg mol^−1^) shows 5× greater flexural strain at break (*ε*_b_ = 3.7%) and 9× higher maximum flexural stress (*σ*_f_ = 72.3 MPa) than the previously accessed medium molar mass samples (24 kg mol^−1^). It is also enchains phthalic anhydride, vinyl cyclohexene oxide and ε-decalactone, *via* switchable catalysis, to make high molar mass triblock polyesters (81 kg mol^−1^, *Đ* = 1.04). This selective catalyst should be used in the future to qualify the properties of these ROCOP polyesters and to tune (multi)block polymer structures.

## Abstract 2

A heterodinuclear Al(iii)/K(i) organometallic ring-opening copolymerization catalyst shows exceptional rates, end-group selectivity and good loading tolerance to deliver monodisperse polyesters with molar masses up to 91 kg mol^−1^.

## Graphical abstract

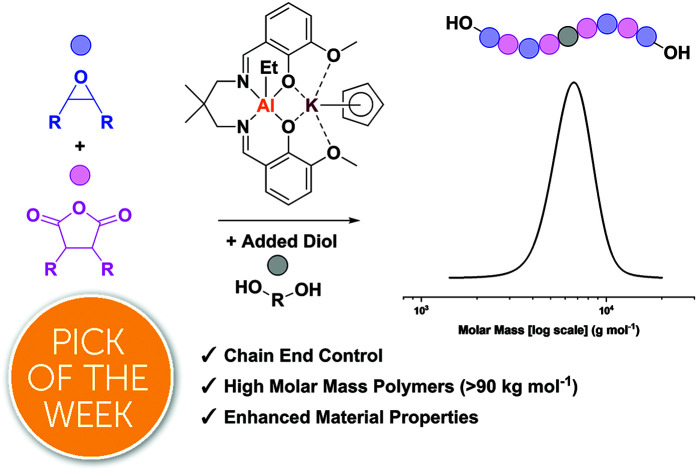

## Introduction

Polyesters are widely used in packaging, consumer goods, clothing, medicine, electronics and construction.^1,2^ Several monomers can be sourced from biomass or wastes and polyesters are often amenable to both mechanical and chemical recycling.^1,2^ Many commercial polyesters are prepared by step-growth methods necessitating condensate removal (high temperature, gas cycling, extended reactions).^3^ These polycondensations are uncontrolled and yield polydisperse products – they are not so suitable for block polymer syntheses. One important chain-growth polymerization is epoxide/anhydride ring-opening copolymerization (ROCOP). It successfully produces aliphatic, semi-aromatic, rigid and functional polyesters.^4,5^ With appropriate catalyst selection it operates with high polymerization control, and tolerates a wide range of monomers, many of which are already commercial products manufactured at scale by the chemical industry and some are bio-based.^4,5^

Over the last decade, significant progress has been achieved in accelerating the catalysis and improving the monomer scope.^4,5^ Metal based catalysts are the highest performing and are usually applied with an ionic co-catalyst.^4–12^ One very successful catalyst design strategy is to covalently attach the co-catalyst to a mononuclear metal complex.^13–15^ Using this approach, a tropylium modified Al(iii)-salen catalyst, reported by Coates and co-workers, for propylene oxide (PO)/phthalic anhydride (PA) ROCOP, showed an excellent turn over frequency (TOF) of 99 h^−1^ at just 0.25 mol% catalyst loading (*vs.* PA, 60 °C).^14^ This year, Lu and co-workers reported an ammonium salt tethered di-Al(iii) salen catalyst for cyclohexene oxide (CHO)/PA ROCOP, which showed TOF of 1437 h^−1^, at 0.25 mol% (*vs.* PA, 100 °C) – it was also highly stereoselective.^16^ The same team also reported an exceptionally active catalyst system, comprising a tri-Cr(iii) complex applied with 3 equiv. of PPNCl for CHO/PA ROCOP, which has a TOF of 10 620 h^−1^ at 0.017 mol% catalyst (*vs.* PA, 100 °C).^17^ Organocatalysts also show promise,^18–20^ with a system comprising an ammonium halide tethered to an organoboron reagent showing a TOF of 258 h^−1^ at 0.5 mol% catalyst for PA/CHO ROCOP (*vs.* PA, 120 °C).^21^ It is also feasible to produce high activity catalysts which operate without ionic co-catalyst, these may be advantageous to simplify catalyst synthesis and obviate corrosive and expensive salts.^22^ In 2021, we reported a heterodinuclear Al(iii)/K(i) complex which operated without a cocatalyst and showed a TOF of 1072 h^−1^ at 0.25 mol% catalyst for PA/CHO ROCOP (*vs.* PA, 100 °C).^23^

**Fig. 1:**
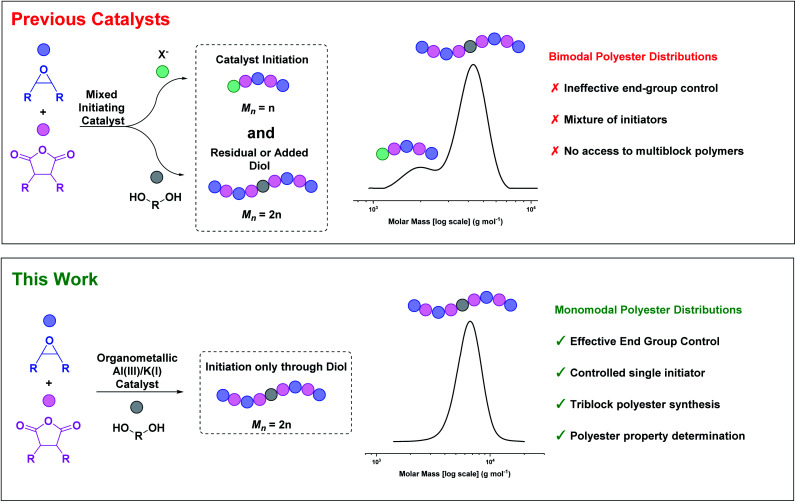
ROCOP catalysts usually have a mixture of initiators resulting in bimodal polyester molar mass distributions.^30^ This work describes an organometallic complex, applied with diol, which yields high molar mass, monomodal polyester distributions.

So far, most catalysts produce low molar mass polyesters, useful as polyols or in surfactant applications.^24–27^ Accessing highly active and tolerant catalysts that produce high molar mass polyesters, with controlled distributions, remains challenging. Whilst a few catalysts can produce high molar mass polyesters, those products typically have bi- or multi-modal molar mass distributions (Fig. 1).^20,21,28^ These polymodal distributions arise because the catalysts comprise different initiators: lower molar mass chains are catalyst or co-catalyst ‘initiated’, while higher molar mass chains are diol/diacid initiated, with these latter species forming by monomer hydrolyses.^29^

The diols/diacids are fast and reversible chain transfer agents (CTA) which means they undergo exchange reactions with catalyst propagating alkoxide/carboxylate chain end groups.^4^ Since all chains initiate at the same rate, the catalyst-initiated chains grow at half the rate of the diol/diacid-initiated chains, forming distinctively bimodal distributions. When targeting low molar mass polymers these issues can be masked, but for higher molar mass polymers they are endemic. The mixed end-groups complicate both the polymerisation reproducibility and proper material thermal–mechanical property evaluation. One common work-around is to suppress catalyst-initiated chain intensity by adding excess CTA (10–20 equiv. *vs.* catalyst),^15,30,31^ but this approach limits the overall polyester molar mass. Another strategy is to maximise the monomer purity by removal of all traces of water or protic compounds, but this route is very challenging since even ppm levels become observable at high molar masses. Rigorous monomer purifications require multiple distillations and necessarily increase the process complexity and energy input. An alternative approach would be to design catalysts that initiate only from the CTA (diol/diacid). We reasoned that the tendency of many organometallic complexes to undergo fast and irreversible reactions with diols/diacid could be exploited to deliver such a catalyst (Fig. 1).

## Results and discussion

**Scheme 1:**
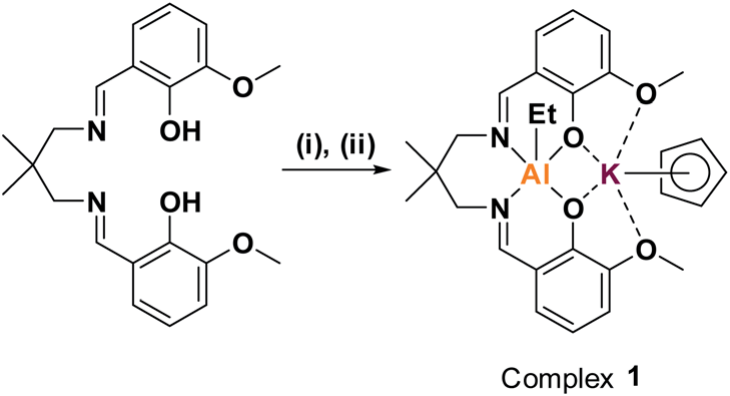
Synthesis of complex 1. (i) 1.05 equiv. AlEt_3_, toluene, RT, 2 h, 64%.^30^ (ii) 1.0 equiv. KCp, THF, RT, 30 m, 99%.

Previously, we reported organometallic Zn(ii)/M(ii) catalysts (M = Zn(ii), Mg(ii)) showing end-group control in epoxide/carbon dioxide ROCOP.^32–34^ Unfortunately, these dinuclear Zn(ii) complexes are not very active in CHO/PA ROCOP (TOF ≈ 25 h^−1^, 1 mol%, 100 °C) and are unreactive using alkylene oxides (*e.g.* PO).^33,35^ We recently reported a heterodinuclear Al(iii)/K(i) catalyst, [L_van_AlK(OAc)_2_], which shows high activity for a wide range of epoxide/anhydride monomers.^23^ The catalyst features acetate initiating groups, so yields polyesters with bimodal molar mass distributions. To properly control end-group chemistry, an organometallic complex was targeted, *i.e.* [L_van_AlK(Cp)(Et)] (1) (Scheme 1).

First, the known organometallic Al(iii) complex, [L_van_AlEt], was produced by reacting the ligand (L_van_H_2_) with triethyl aluminium, in toluene, and was isolated in 64% yield.^30^ Next, an organo-potassium reagent, KCp, was added to a solution of [L_van_AlEt], in THF, resulting in an immediate colour change from yellow to pale orange.^30^ After removal of the reaction solvent, the target organometallic complex was isolated in quantitative yield. Complex 1 was characterized by solid state IR and solution-phase NMR spectroscopy (Fig. S1–S6†). The ^1^H NMR spectrum shows shifts to the Al-ethyl resonances, indicative of increased anionic character, after K(i) coordination. A single resonance, at 5.60 ppm, is observed for the Cp protons indicative of η^5^-K(i) coordination and its integrals are in the expected 1 : 1 stoichiometry *vs.* both the ligand (L_van_) and ethyl moieties. The L_van_ methylene and methyl protons are diastereotopic, giving rise to a set of two resonances each. The ^13^C{^1^H} NMR spectrum is also consistent with the proposed structure, as are elemental analyses. The ^27^Al NMR spectrum does not show a signal which is consistent with a pentacoordinate Al(iii) centre.

When applied in catalysis, 1 reacts with a diol CTA to form an alkoxide initiator *in situ* (*vide infra*). To investigate this alcoholysis process, 1 was reacted with 2 equiv. of 4-fluorophenol, in THF, at room temperature. The resultant Al(iii)/K(i) aryloxide complex (2, see ESI† for structure) displayed new resonances corresponding to two coordinated 4-fluorophenolates, and the complete disappearance of resonances of both organometallic ligands (Fig. S7–S10†). Its ^27^Al NMR spectrum shows a sharp peak, at 1.6 ppm, consistent with octahedral Al(iii). Thus, the Al(iii)/K(i) aryloxide complex is proposed to have an ‘aluminate’ centre, with a cationic K^+^ coordinated by the ether groups. This finding could be significant to the polymerization mechanism, particularly as the intermediate catalyst-alkoxide moieties are rarely structurally characterized in this field of catalysis.^36^

**Fig. 2:**
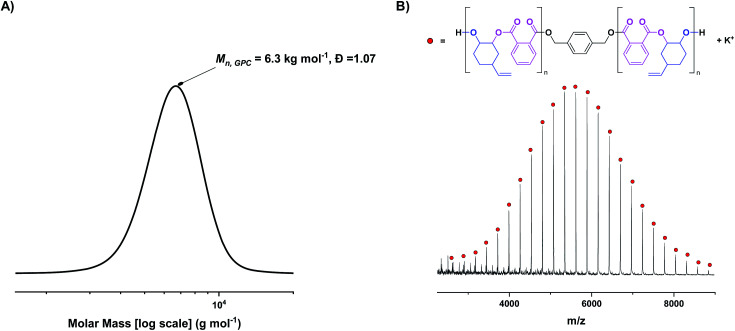
(A) Molar mass distribution of poly(vinyl cyclohexene phthalate) (PvCHPE) obtained with complex 1. Conditions: [1] : [BDM] : [PA] : [vCHO] = 1 : 4 : 100 : 2000, *T* = 100 °C. (B) MALDI-ToF spectrum of PvCHPE obtained. *M*_n,calc_ (repeat unit) = 272.3 g mol^−1^, *M*_n,theoretical_ (repeat unit) = 272.3 g mol^−1^; *M*_n,calc_ (end group = BDM + K^+^) = 177.4 g mol^−1^, *M*_n,theoretical_ (end group) = 177.3 g mol^−1^.

Complex 1 was tested in the ROCOP of vinyl cyclohexene oxide (vCHO) and PA, using 4 equiv. of 1,4-benzene dimethanol (1,4-BDM) as the initiator/CTA. Initially, high catalyst loadings were used (1 mol% *vs.* anhydride), forming low molar mass polyesters suitable for end-group analysis. GPC analysis shows the polyester has a monomodal, narrow molar mass distribution (*Đ* = 1.07) with a molar mass value (6 kg mol^−1^) close to that expected theoretically (Fig. 2A). MALDI-ToF mass spectrometry shows a single series of peaks, consistent with chains only being initiated from 1,4-BDM, *i.e.* formation of only telechelic α,ω-hydroxyl-polyester (Fig. 2B). Under equivalent conditions, [L_van_AlK(OAc)_2_] yields bimodal polymer molar mass distributions, featuring both α,ω-hydroxy- and α-acetate-ω-hydroxy polyesters (Fig. S11†).^23^

To test its generality, a range of terminal and internal epoxides were copolymerized with PA (Table 1, Fig. S12–S21†). In all experiments, a low catalyst loading (0.25 mol% *vs.* anhydride; 0.042 mol% overall) was employed to access higher molar mass polyesters, and to demonstrate catalyst applicability under challenging conditions. Catalyst 1 was highly active with TOF values from 100–1000 h^−1^ (*T* = 100 °C); such values are at the upper end of this field.^14,17,37^ It also generally showed quantitative ester linkage selectivity and, importantly, all the polyesters show monomodal molar mass distributions with narrow dispersity (*Đ* < 1.10). High activities were maintained using both internal (CHO/PA : TOF = 1032 h^−1^; vCHO/PA : TOF = 528 h^−1^) and terminal epoxides (*tert*-butyl glycidyl ether (*t*BGE)/PA : TOF = 324 h^−1^; allyl glycidyl ether (AGE)/PA : TOF = 280 h^−1^; PO/PA : TOF = 17 h^−1^) (Table 1).

**Table 1:** Data for the ROCOP of PA and various epoxides using catalyst 1^a^

Entry	Epoxide	Temperature (°C)	Ester selectivity^b^ (%)	TOF^c^ (h^−1^)	*M*_n,GPC_ d (kg mol^−1^)	*Đ* e	*M*_n,Th._ f (kg mol^−1^)	DP_NMR_^g^
1	CHO	100	95^h^	1032	20.1	1.10	24.8	104
2	vCHO	100	>99	528	24.8	1.05	27.4	N.d.^i^
3	*t*BGE	100	>99	324	19.6	1.10	28.0	99
4	AGE	100	>99	280	16.5	1.10	25.0	97
5	PO	60	>99	17	18.6	1.07	20.8	100

a General conditions: [1] : [1,4-BDM] : [PA] : [epoxide] = 1 : 4 : 400 : 2000. All reactions run to >99% anhydride conversion.

b Selectivity for ester over ether linkages, determined by ^1^H NMR spectroscopy.

c TOF = TON/time (hours). Estimated from aliquots taken during reaction, see Table S1 for details.

d Determined by gel permeation chromatography (GPC) in tetrahydrofuran, at 30 °C, using narrow dispersity polystyrene standards.

e Dispersity = *M*_w_/*M*_n_, determined by GPC in tetrahydrofuran, at 30 °C.

f Theoretical molar mass, determined by (TON × *M*_n,repeat unit_/4) + *M*_n,BDM_.

g Determined by ^1^H NMR spectroscopy by through integral analysis of the BDM *vs.* polyester resonances.

h Low levels (∼5%) of ether production are attributed to Cp-moieties, as [L_van_AlK(OAc)_2_] produces no ether under analogous conditions.^23^

i Not determined due to peak overlap in ^1^H NMR spectrum.

The theoretical and experimental molar masses (GPC) were in reasonable agreement, as were the expected and experimental degrees of polymerization (DP, determined by ^1^H NMR spectroscopy by integration of end-group *vs.* main chain signals; DP_theoretical_ = 100). The polyesters synthesized span rigid plastics (CHO/PA), elastomers (*t*BGE/PA, PO/PA) and vinyl-functionalized materials (vCHO/PA, AGE/PA) which can undergo post-polymerization reactions to modify properties.^20,38^ Previously, rigid, high *T*_g_ polyesters, such as those derived from PA/CHO, or PA/vCHO, were used with low *T*_g_ aliphatic polyesters to prepare block polymer thermoplastic elastomers.^33,39,40^ Such block polyesters may be efficiently synthesized using catalysts that selectively enchain mixtures of epoxide, anhydride and lactone.^35,41,42^ To test the potential for complex 1 in such switchable catalysis, the one pot polymerization of PA, vCHO and ε-decalactone (DL) was investigated (Table S2, Fig. S22 and S23†). Catalyst 1 showed exceptional control, selectively producing a triblock polymer with a molar mass of 80.8 kg mol^−1^ and low dispersity (*Đ* = 1.04). The resultant triblock polyester possessed 30 wt% hard block content, with DP values of PDL_184_–PvCHPE_100_–PDL_184_, as determined by ^1^H NMR spectroscopy. Aliquot analysis confirmed that complete anhydride consumption occurred before lactone conversion began, consistent with the rules of switchable catalysis and ensuring clean formation of triblock polyester.^42^ Catalyst 1 shows a good activity (TOF = 150 h^−1^, 0.0625% *vs.* lactone, *T* = 100 °C) for DL ROP under these conditions.

To investigate the unprecedented polymerization control afforded by catalyst 1, a series of vCHO/PA polymerizations were undertaken at progressively lower catalyst loadings (Table 2). All reactions were performed on gram-scale to facilitate subsequent material testing (*vide infra*). At this increased scale, slightly lower activities (TOF *ca.* 400 h^−1^) were obtained, which is attributed to unoptimized stirring in glassware (viscosity limitations). Nonetheless, the activity remained constant even at very low loadings, consistent with its high tolerance (0.04% *vs.* anhydride, 0.007% overall, Table 2, entry 4). All the reactions are very well controlled, forming polyesters with monomodal molar mass distributions and low dispersities (*Đ* < 1.08). There is an increase in molar mass with reduced catalyst loading, reaching a value of 91 kg mol^−1^ at the lowest loading investigated (Table 2, entries 1–4). At higher molar mass there is some divergence between the predicted and experimental molar masses. This finding is quite common in other polymerizations,^21,43^ particularly at such low catalyst loadings, and probably arises from residual CTA either in the monomers or from experimental set-up. After accounting for the low levels of residual CTA, there is excellent agreement between experimental and calculated molar masses at all loadings (Table 2, Fig. S25†).

**Table 2:** Data for gram-scale PA/vCHO ROCOP with catalyst 1^a^

Entry	Name^b^	[Cat] : [PA]	*M*_n,GPC_ c (kg mol^−1^)	*Đ* d	*M*_n,Th._ e (kg mol^−1^)	*M*_n,Th.Adj._ f (kg mol^−1^)	DP_Exptl._^g^
1	PvCHPE-24	400	23.5	1.06	27.3	24.2	86
2	PvCHPE-44	800	43.5	1.06	54.6	43.6	160
3	PvCHPE-70	1600	70.1	1.07	109.0	72.6	257
4	PvCHPE-91	2400	91.0	1.08	163.5	93.4	334

a Conditions: [1] : [BDM] : [PA] : [epoxide] = 1 : 4 : *x* : *y* where *x* is given and *x* : *y* = 1 : 5, *T* = 100 °C. All reactions run to >99% anhydride conversion.

b Naming convention: ester acronym-molar mass (kg mol^−1^).

c Determined by gel permeation chromatography (GPC) in tetrahydrofuran, at 30 °C, using narrow dispersity polystyrene standards.

d Dispersity = *M*_w_/*M*_n_, determined by GPC in tetrahydrofuran, at 30 °C.

e Theoretical molar mass, determined by (TON × *M*_n,repeat unit_/4) + *M*_n,BDM_.

f Theoretical molar mass accounting for residual chain transfer agent (calculated as [CTA]_residual_ : [PA] : [vCHO] = 1 : 800 : 4000, see Fig. S25).

g Degree of polymerization, determined by *M*_n,GPC_/*M*_n,repeat unit_. Note that, due to the high DP values, NMR measurements are not appropriate.

Prior access to high molar mass ROCOP polyesters is very limited and, where the GPC traces are reported, nearly all show significantly bimodal distributions (*vide supra*).^16,20,21,28,44^ For example, the ammonium halide tethered organoborane produced poly(PA-*alt*-CHO) with *M*_n_ of 95 kg mol^−1^ but with a ∼50 : 50 distribution of mono and bifunctional chains.^21^ The tethered Al(iii)salen and tri-Cr(iii)/PPNCl catalysts referred to earlier both showed excellent loading tolerance but also yield polyesters with bimodal molar mass distributions.^14,17^ There is just one report of an organocatalyst, ^*t*^BuP_1_, that produces monodisperse PA-*alt*-CHO and PA-*alt*-vCHO, although the molar masses were low/moderate at *M*_n_ < 30 kg mol^−1^.^20^ In comparison to these leading catalysts, complex 1 combines high rates and excellent loading tolerance with exceptional end group control. These features ensure that all the polyesters are monodisperse even at very low catalyst loadings. These polyesters are suitable for thermal–mechanical property characterization; such important data have historically been under-reported due to the very low molar masses obtained and the inhomogeneous polymer structures.^20^ Notably, previously produced polyesters show very distinct bimodal molar mass distributions, which impact any material property measurements.

**Fig. 3:**
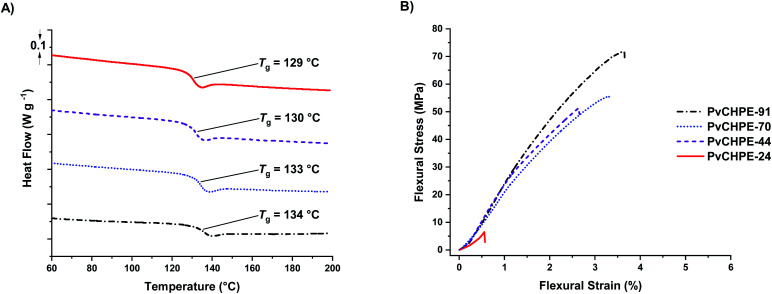
Thermal and mechanical data for PvCHPE samples (Table 2). (A) DSC data shows the increase in *T*_g_ with molar mass. (B) Representative bending stress–strain data for PvCHPE samples (DMA; 3-point bend method, 0.1% s min^−1^, 25 °C).

The series of monomodal PvCHPE samples were investigated by differential scanning calorimetry (DSC). All samples are amorphous and there was an increase in *T*_g_ (129–134 °C) with increasing molar mass (Table 3, entries 1–4, Fig. 3A). Thermogravimetric analyses show that all polyesters have a high on-set thermal decomposition temperatures (*T*_d,5%_) of *ca.* 320 °C, hence establishing a polymer processing window of *ca.* 180 °C (Fig. S26–S29†). The samples were processed *via* hot press methods to yield transparent bars suitable for dynamic mechanical–thermal analysis (DMTA).

**Table 3:** Thermal and mechanical data for PvCHPE samples reported in Table 2

Entry	Name	*T*_g,DSC_ a (°C)	*ε*_b_ b (%)	*σ*_f_ c (MPa)	*E*_f_ d (GPa)
1	PvCHPE-24	129	0.7 ± 0.1	8.2 ± 1.3	1.6 ± 0.3^e^
2	PvCHPE-44	130	2.4 ± 0.1	49.1 ± 2.9	2.6 ± 0.2
3	PvCHPE-70	133	3.0 ± 0.1	61.4 ± 3.4	2.8 ± 0.1
4	PvCHPE-91	134	3.7 ± 0.2	72.3 ± 3.3	3.0 ± 0.2

a Glass transition temperature, measured by DSC, as midpoint of transition during second heating cycle.

b Flexural strain at break, determined by 3-point bend DMA (0.1% s min^−1^, 25 °C). See ESI for calculation details.

c Ultimate flexural strength, determined by 3-point bend DMA (0.1% s min^−1^, 25 °C). See ESI for calculation details.

d Flexural modulus from DMA as the gradient of stress/strain data from 0.5–1% strain.

e Modulus estimated between 0.2–0.5% strain.

Both tension film and 3-point bend experiments were undertaken, although, the lowest molar mass sample (PvCHPE-24) was brittle and shattered before being tested for the tension film methodology. There was no change in polymer structure during processing, as confirmed by ^1^H NMR spectroscopy and GPC characterization of samples before and after processing (Fig. S30 and S31†). The tension film DMA measurements showed the polyesters have high storage (>1 GPa), and low loss (<100 MPa) moduli (Table S3, Fig. S32–S34†). The best material had the highest molar mass with PvCHPE-91 showing a storage modulus of 2.14 GPa, 20% higher than the equivalent value for PvCHPE-44 (Table S3, entries 2 and 4†). The 3-point bend experiments also demonstrated the benefits of producing pure high molar mass polyesters (Table 3, entries 1–4, Fig. 3B). The lowest mass polymer, PvCHPE-24 is both weak and brittle, with a flexural strain at break (*ε*_b_) of just 0.7 ± 0.1%, maximum flexural strength (*σ*_f_) of 8.2 ± 1.3 MPa, and a flexural modulus (*E*_f_) 1.6 GPa (Table 3, entry 1). As the molar mass increases, so the properties improve and the best results are obtained for PvCHPE-91 (*ε*_b_ = 3.7 ± 0.2% and *σ*_f_ = 72.3 ± 3.3 MPa and *E*_f_ = 3.0 GPa). PvCHPE-91 shows 5× greater elasticity, 9× higher ultimate flexural strength and 2× higher flexural modulus compared to lower molar mass samples. These findings suggest that chain entanglement occurs between 24 and 44 kg mol^−1^. PvCHPE-91 has comparable tensile mechanical properties to polystyrene (*ε*_b_ = 1.6%, *σ*_f_ = 43 MPa, *G*′ = 3 GPa), bis-phenol A derived polycarbonate (*ε*_yield_ = 3.5%, *σ* = 50 MPa, *G*′ = 2.1 GPa) or polyethylene terephthalate (*ε*_yield_ = 3.5%, *σ* = 55 MPa, *G*′ = 2.3 GPa) although, in comparison to the latter two polymers, it does not display a yield point.^45^ The ability of the PvCHPE polyester to match some of the tensile mechanical properties of currently industrialized polymers is interesting, and future investigations of these new high molar mass polyesters to develop their properties and applications are warranted.

## Conclusions

An organometallic Al(iii)/K(i) catalyst system shows high activity, selectivity and end-group control in epoxide/anhydride ROCOP. It produces high molar mass, hydroxyl telechelic polyesters with monomodal, monodisperse distributions. The complex reacts stoichiometrically with alcohols and an isolated aryloxide complex is a putative catalytic cycle intermediate. It successfully enchains internal, external and vinyl-functionalised epoxides producing polyesters with interesting thermal–mechanical properties. As proof of potential, an amorphous, high *T*_g_ polyester shows significantly better tensile mechanical properties than previously reported materials as a result of its higher molar mass. The catalyst was also used, in one-pot, to enchain mixtures of epoxide, anhydride and lactone to produce high molar mass triblock polyester. In future, this end-group control should be used to properly quantify the properties of ROCOP polyesters and to make high molar mass multi-block polymers.^42,46,47^

## Data availability

Experimental procedures, complex characterization data (NMR, IR and elemental analysis) and further polymerisation data (GPC chromatograms, NMR and MALDI-ToF spectra, DMTA plots) are available in the ESI.†

## Author contributions

WD carried out all experimental procedures. CW and WD wrote the manuscript.

## Conflicts of interest

The authors declare no competing financial interest.

## Supplementary Material

SC-013-D2SC02752F-s001
