# Supplementary material for: Chain end-group selectivity using an organometallic Al(iii)/K(i) ring-opening copolymerization catalyst delivers high molar mass, monodisperse polyesters
Source: Chem Sci. 2022 Jul 13;13(29):8543–9. doi: 10.1039/d2sc02752f (PMC9337735; doi:10.1039/d2sc02752f)
Supplement: SC-013-D2SC02752F-s001 [file SC-013-D2SC02752F-s001.pdf]

Electronic Supplementary Material (ESI) for Chemical Science.
This journal is © The Royal Society of Chemistry 2022

Electronic Supporting Information

For

Chain End-Group Selectivity Using an Organometallic Al(III)/K(I) Ring-Opening Copolymerization Catalyst Delivers High Molar Mass, Monodisperse Polyesters

Wilfred T. Diment, Charlotte K. Williams*

Chemistry Research Laboratory, Department of Chemistry,

University of Oxford, 12 Mansfield Road, Oxford, OX1 3TA, UK.

General Procedures and Materials 4
Methods 5
Synthesis of Complexes 7
Representative Procedures 10
Figure S1. ^{1}H NMR spectrum (400 MHz, 298 K, THF-d_8(*)) of complex **1**. 12
Figure S2. ^{13}C NMR spectrum (126 MHz, 298 K, THF-d8(*)) of complex **1** 12
Figure S3. ^{1}H COSY NMR Spectrum (400 MHz, 298 K, THF-d_8) of complex **1**. 13
Figure S4. ^{1}H-^{13}C HSQC NMR Spectrum (298 K, THF-d_8) of complex **1**. 13
Figure S5. ^{1}H-^{13}C HMBC NMR Spectrum (298 K, THF-d_8) of complex **1**. 14
Figure S6. Infrared spectrum of complex **1**. 14
Figure S7. ^{1}H NMR spectrum (400 MHz, 298 K, THF-d_8) of complex **2**. 15
Figure S8. ^{1}H COSY NMR spectrum (400 MHz, 298 K, THF-d_8) of complex **2**. 15
Figure S9. ^{27}Al NMR spectrum (104 MHz, 298 K, THF-d_8) of complex **2**. 16
Figure S10. ^{19}F NMR spectrum (THF-d_8, 376 MHz, 298K) of complex **2** 16
Figure S11. PvCHPE synthesised with [L_{van}AlK(OAc)$_2$] 17
Table S1. Aliquot data used to determine rates of epoxide/PA ROCOP with complex **1**.[a] 17
Figure S12. GPC trace of poly(CHO-*alt*-PA) obtained with complex **1**, ... 18
Figure S13. ^{1}H NMR spectrum ($CDCl_3$, 400 MHz, 298K) of isolated poly(CHO-*alt*-PA). 18
Figure S14. GPC trace of poly(vCHO-*alt*-PA) (PvCHPE) obtained with complex **1**, ... 19
Figure S15. ^{1}H NMR spectrum ($CDCl_3$, 400 MHz, 298K) of isolated poly(vCHO-*alt*-PA). 19
Figure S16. GPC trace of poly(tBGE-*alt*-PA) obtained with complex **1**, ... 20
Figure S17. ^{1}H NMR spectrum ($CDCl_3$, 400 MHz, 298K) of isolated poly(tBGE-*alt*-PA). 20
Figure S18. GPC trace of poly(AGE-*alt*-PA) obtained with complex **1**, ... 21
Figure S19. ^{1}H NMR spectrum ($CDCl_3$, 400 MHz, 298K) of isolated poly(AGE-*alt*-PA). 21
Figure S20. GPC trace of poly(PO-*alt*-PA) obtained with complex **1**, ... 22
Figure S21. ^{1}H NMR spectrum ($CDCl_3$, 400 MHz, 298K) of isolated poly(PO-*alt*-PA). 22
Table S2. Synthesis of a triblock copolymer with complex **1**.[a] 23
Figure S22. GPC Traces of aliquots taken during triblock synthesis, ... 23
Figure S23. ^{1}H NMR spectrum ($CDCl_3$, 400 MHz, 298 K) of isolated PDL-*b*-PvCHPE-*b*-PDL synthesised with complex **1**. 24
Figure S24. GPC Traces of PvCHPE-24, -44, -70 and -91, corresponding to Table 2, Entries 1 – 4, respectively. 25
Figure S25. Calculation of residual CTA, based on Table 2, Entry 2. 25
Figure S26. TGA Trace for PvCHPE-24 26
Figure S27. TGA Trace for PvCHPE-44 26
Figure S28. TGA Trace for PvCHPE-70 27

Figure S29. TGA Trace for PvCHPE-91 27

Figure S30. Typical GPC Trace of PvCHPE before and after processing *via* hot press (shown: PvCHPE-24). 28

Figure S31. ^{1}H NMR ($CDCl_3$, 400 MHz, 298 K) of isolated PvCHPE-24 before and after processing *via* hot press. 28

Table S3. Mechanical Data for PvCHPE series analysed by Tension Film DMTA. 29

Figure S32. . Representative data from DMTA analysis of PvCHPE-44 *via* tension film methodology, between 30 – 200 °C. Storage Modulus (E'), Loss Modulus (E''), and Tan(δ) presented. 29

Figure S33. Representative data from DMTA analysis of PvCHPE-70 *via* tension film methodology, between 30 – 200 °C. Storage Modulus (E'), Loss Modulus (E''), and Tan(δ) presented. 29

Figure S34. Representative data from DMTA analysis of PvCHPE-91 *via* tension film methodology, between 30 – 200 °C. Storage Modulus (E'), Loss Modulus (E''), and Tan(δ) presented. 30

References 31

General Procedures and Materials

All synthetic manipulations were carried out in a nitrogen filled glovebox, or a dual manifold nitrogen-vacuum Schlenk line. Solvents used in synthesis were collected from a solvent purification system (SPS), degassed with three freeze-pump-thaw cycles and stored over 3 Å molecular sieves under an inert atmosphere, unless otherwise stated. All materials were stored under a nitrogen atmosphere, in a glovebox. Triethyl Aluminium (93%) was purchased from Sigma Aldrich and was used as received. 4-Fluorophenol was purchased from Sigma Aldrich and was sublimed at room temperature before use. Potassium cyclopentadiene was synthesised following a literature procedure.[1]

Vinyl cyclohexene oxide (vCHO, Sigma Aldrich), propylene oxide (PO, Sigma Alrich), *tert*-butyl glycidyl ether (tBGE, Sigma Aldrich) were purified by stirring over calcium hydride, followed by fractional distillation. Cyclohexene oxide (CHO, Acros Organics) and allyl glycidyl ether (AGE, Sigma Aldrich) were purified by a three-fold distillation process. Firstly, the epoxide was stirred over calcium hydride for 16 hours and subsequently fractionally distilled. The distillate was then stirred with nBuLi in hexanes (5 v/v%) for 16 hours, and the mixture was subsequently fractionally distilled under reduced pressure. A final, subsequent, distillation of the collected fraction was performed under reduced pressure. Phthalic anhydride (PA, Sigma Aldrich) was purified though a three-step procedure. Firstly, PA was stirred in dry toluene at room temperature for 16 hours. The supernatant was filtered, and the toluene subsequently removed *in vacuo*. The resultant white powder recrystallised from hot (60 °C) chloroform and subsequently sublimed under vacuum at 80 °C.

The pro-ligand, [$L_{van}H_2$], was synthesised following a literature procedure.[2]

Methods

NMR Spectroscopy: ^{1}H NMR and ^{27}Al spectra were obtained using a Bruker Avance III HD 400 NMR spectrometer. ^{13}C{^{1}H} NMR were obtained with either Bruker AV III HD 500 MHz NMR, Bruker NEO 600 MHz with broadband helium cryprobe, or Bruker AVIII 700 MHz with inverse TCI 1H/13C/15N cryoprobe spectrometers.

IR Spectroscopy: Carried out on a Shimadzu IRSpirit FT-IR spectrometer equipped with a single reflection ATR (attenuated total reflectance) accessory, in a nitrogen filled glovebox.

GPC analysis: Carried out using a Shimadzu LC-20AD instrument, equipped with a Refractive Index (RI) detector and two PSS SDV 5 µm linear M columns. The eluent used was HPLC-grade THF, heated to 30 °C, and with a flow rate of 1.0 mL min^{-1}.

MALDI-ToF spectrometry: Carried out on a Bruker Autoflex Speed MALDI-ToF spectrometer. Samples were prepared by preparing a 1:4:1 solution of polymer (10 mg mL^{-1} in THF), dithranol (10 mg mL^{-1} in THF) and KTFA (10 mg mL^{-1} in THF). This solution was spotted twice on a MALDI plate and allowed to dry completely before analysis was undertaken.

Elemental analysis was carried out by London Metropolitan University Elemental Analysis Service.

Differential Scanning Calorimetry (DSC): Carried out using a DSC 25 (TA Instruments). Samples were heated to 200 °C for 5 minutes to remove thermal history, before heating and cooling from 30 °C to 200 °C at a rate of 10 °C min^{-1}. Glass transition temperatures (T_g) were recorded from the midpoint of the transition during the third heating curve.

Thermal Gravimetric Analysis (TGA): Carried out using a TGA/DSC 1 (Mettler-Toledo Ltd.) instrument. Samples were heated from 30 °C to 500 °C, under continuous N_2 flow, at a rate of 20 °C min^{-1}.

Dynamic Mechanical Thermal Analysis (DMTA): Carried out on a DMA 850 (TA Instruments). Suitable samples were prepared *via* hot press (see representative procedures, below). For tension film experiments the following procedure was applied: samples were heated from 30 °C to 180 °C at 3 °C min^{-1} and oscillated at a frequency of 1 Hz with 0.05% strain. For 3-point bend experiments the following procedure was applied: samples were subjected to a strain ramp, at 25 °C, from 0% to break at 0.1% strain min^{-1}.

To calculate flexural stress and strain from 3-point bend experiments, the following formulae were applied and calculated using the TRIOS software package (TA Instruments):

$$\sigma = \frac{3PL}{wt^2}$$

$$\varepsilon = \frac{6\delta t F_C}{L^2[1 + \frac{12}{5}(1+\nu)\left(\frac{t}{L}\right)^2]}$$

Where:

σ = stress

ε = strain

P = ½ applied force

δ = amplitude of deformation

L = sample length (span)

t = sample thickness

w = sample width

F_c = clamping correction factor

ν = Poisson's Ratio (taken to be 0.45)

Synthesis of Complexes

Synthesis of L_{van}AlEt

This complex was synthesised following an adapted literature procedure.[3] Under inert conditions, $AlEt_3$ (0.18 g, 1.6 mmol) was added to a stirring solution of [$L_{van}H_2$] (0.53 g, 1.4 mmol) in anhydrous toluene (10 mL). The reaction mixture was stirred for 1 hour. A yellow powder precipitated from the reaction mixture, which was isolated *via* centrifuge, washed with toluene (3 x 10 mL) and hexane (1 x 10 mL) and subsequently dried under vacuum to give [L_{van}AlEt] as a yellow powder (0.38 g, 0.9 mmol, 64%). The spectra of the complex were consistent with those previously reported.

Synthesis of L_{van}AlK(Cp)(Et) (1)

Under inert conditions, KCp (18 mg, 0.17 mmol) was added to a stirred solution of complex [L_{van}AlEt] (73 mg, 0.17 mmol) in anhydrous THF at room temperature, and was stirred for 30 minutes. Subsequently the solvent was removed under vacuum to give **1** as a pale orange powder (101 mg, 0.17 mmol, 99%). The complex was stored at -30 °C, under an inert atmosphere.

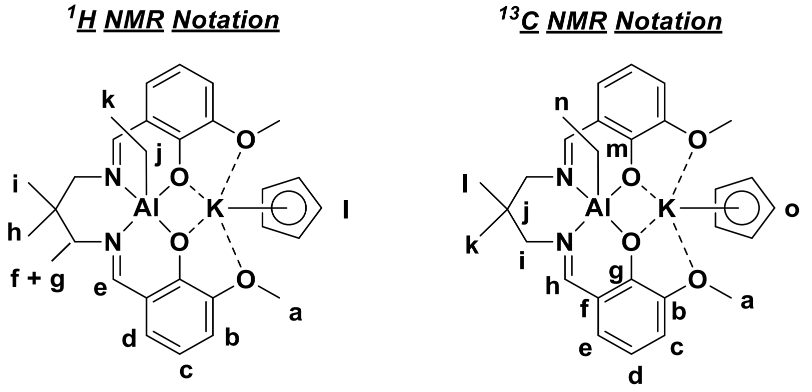

^{1}H NMR: (THF-d_8, 400 MHz, 298 K): δ 8.25 (2H, s, H^e), 6.96 (2H, dd, *J* = 7.8, 1.4 Hz, H^b), 6.91 (2H, dd, *J* = 7.8, 1.4 Hz, H^d), 6.62 (2H, t, *J* = 7.8 Hz, H^c), 5.60 (5H, s, H^l), 3.89 (6H, s, H^a), 3.63

(2H, d, J = 12.2 Hz, **^{13}C NMR:** (THF-d_8, 126 MHz, 298 K): δ 171.6 (C^h), 157.1 (C^f), 152.3 (C^b), 125.9 (C^e), 120.4 (C^g), 117.5 (C^c), 116.6 (C^d), 104.7 (C^o), 69.3 (C^i), 56.9 (C^a), 36.7 (C^j), 26.4(C^k), 24.6 (C^l), 10.6 (C^n), 7.0 (C^m) ppm. **IR**: 347, 356, 363, 370, 379, 392, 460, 477, 512, 626, 649, 701, 739, 859, 976, 1005, 1069, 1082, 1169, 1198, 1231, 1247, 1304, 1340, 1391, 1401, 1452, 1480, 1555, 1615, 2793, 2855, 2928, 3055 cm^{-1}. **Anal. Calc.** ($C_{28}H_{34}AlKN_2O_4$·0.5(C_4H_8O)); C, 63.81; H, 6.78; N, 4.96 %. Found: C, 64.21; H, 6.54; N, 4.52 %. (*N.b.* ½ an equivalent of THF is present in the complex, as confirmed by ^{1}H NMR spectroscopy; the sample cannot be dried to completeness due to decomposition under vacuum over extended periods (>1 hour)).

Synthesis of $L_{van}AlK(O(C_6H_4)F)_2$ (2)

Under inert conditions, 4-fluorophenol (5.1 mg, 0.4 mmol) was added to a stirred solution of **1** (12 mg, 0.2 mmol) in THF (3 mL) at room temperature. An immediate colour change from pale orange to pale yellow was observed. The solution was stirred for 30 minutes, before the solvent was remove under vacuum yielding **2** as a pale-yellow powder (14.4 mg, 0.2 mmol, 99%).

^{1}H NMR Notation

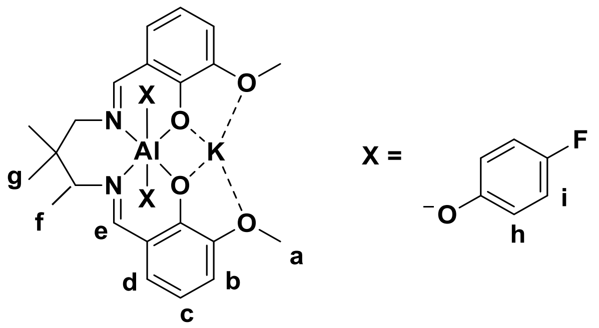

^{1}H NMR: (THF-d_8, 400 MHz, 298 K): δ 7.91 (2H, s, H^e), 6.72 (4H, m, H^d and H^b), 6.35 (2H, t, J = 7.7 Hz), 6.27 (4H, m, H^h or H^i), 6.06 (4H, m, H^h or H^i), 3.76 (6H, s, H^a), 3.51 (4H, s, H^f), 1.12 (6H, s, H^g) ppm. **^{19}F NMR:** (THF-d_8, 376 MHz, 298K): δ -131.7 ppm. **^{27}Al NMR:** (THF-d_8, 104 MHz, 298K): δ 0.85 ppm.

Representative Procedures

Epoxide/Anhydride Copolymerization

Inside a nitrogen filled glovebox, complex **1**, 1,4-benzenedimethanol (1,4-BDM), and the stated epoxide and anhydride monomers were added to a dried vial, equipped with a magnetic stirrer bar, in the ratios specified (typically [**1**]:[BDM]:[Anh]:[Ep] = 1:4:400:2000). The vial was sealed with a melamine-cap containing a Teflon inlay, and further sealed with first Parafilm M and then electrical insulation tape. This sealed vial was then heated to the stated temperature for the stated time. Aliquoting was performed by first cooling the polymerisation vial, in a cold-water bath, before removing ca. 10 μL of the polymerisation mixture with a syringe in the glovebox. The polymerisations were quenched by exposing the reaction mixture to air, followed by removal of volatiles. A ^{1}H NMR spectrum of the crude product was measured in $CDCl_3$. GPC samples were prepared by dissolving ca. 10 mg of the crude product in 1 mL HPLC grade THF and filtered before use. Polymers were purified by precipitation from methanol and dried under vacuum at 40 °C.

Switchable Polymerization of Epoxide/Anhydride/Lactone

Inside a nitrogen filled glovebox, complex **1**, 1,4-BDM, and the stated epoxide, anhydride and lactone monomers were added to a dried vial, equipped with a magnetic stirrer bar, in the ratios specified. The vial was sealed with a melamine-cap containing a Teflon inlay, and further sealed with first Parafilm M and then electrical insulation tape. This sealed vial was then heated to 100 °C for the time stated. Aliquoting was performed by first cooling the polymerisation vial, in a cold water bath, before removing ca. 10 μL of the polymerisation mixture with a syringe in the glovebox. The polymerizations were quenched by exposing the reaction mixture to air, followed by removal of volatiles. A ^{1}H NMR spectrum of the crude

product was measured in $CDCl_3$. GPC samples were prepared by dissolving ca. 10 mg of the crude product in 1 mL HPLC grade THF and filtered before use. Polymers were purified by precipitation from methanol and dried under vacuum at 40 °C.

Sample Preparation for Mechanical Analysis

A dried powder sample of the desired poly(vinyl cyclohexene phthalate) (PvCHPE) was placed in a mould and subjected to 140 °C and 4 metric tonnes of pressure until a clear film was obtained (~ 30 minutes). From these films, bars suitable for mechanical analysis were cut using a scalpel. Typical dimensions *ca*. 15 mm * 5 mm * 0.3 mm [lwd]).

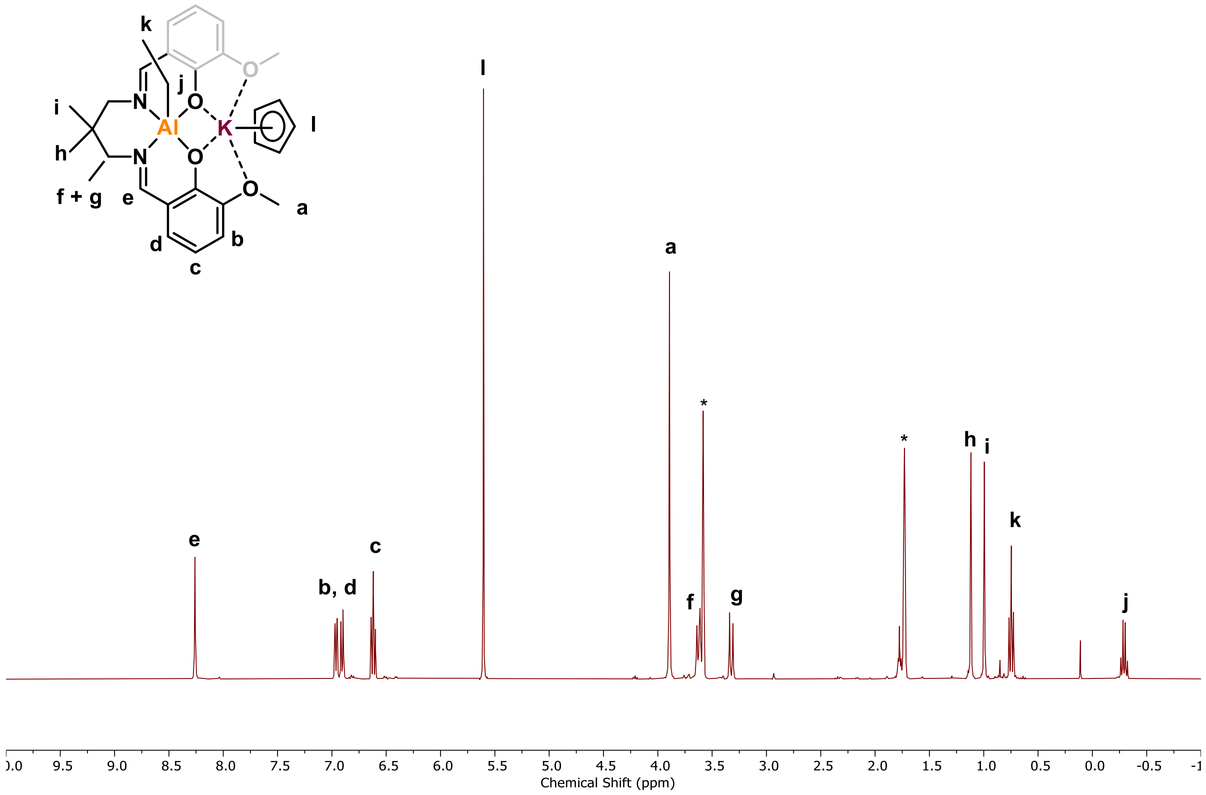

Figure S1. ^{1}H NMR spectrum (400 MHz, 298 K, THF-d_8(*)) of complex **1**.

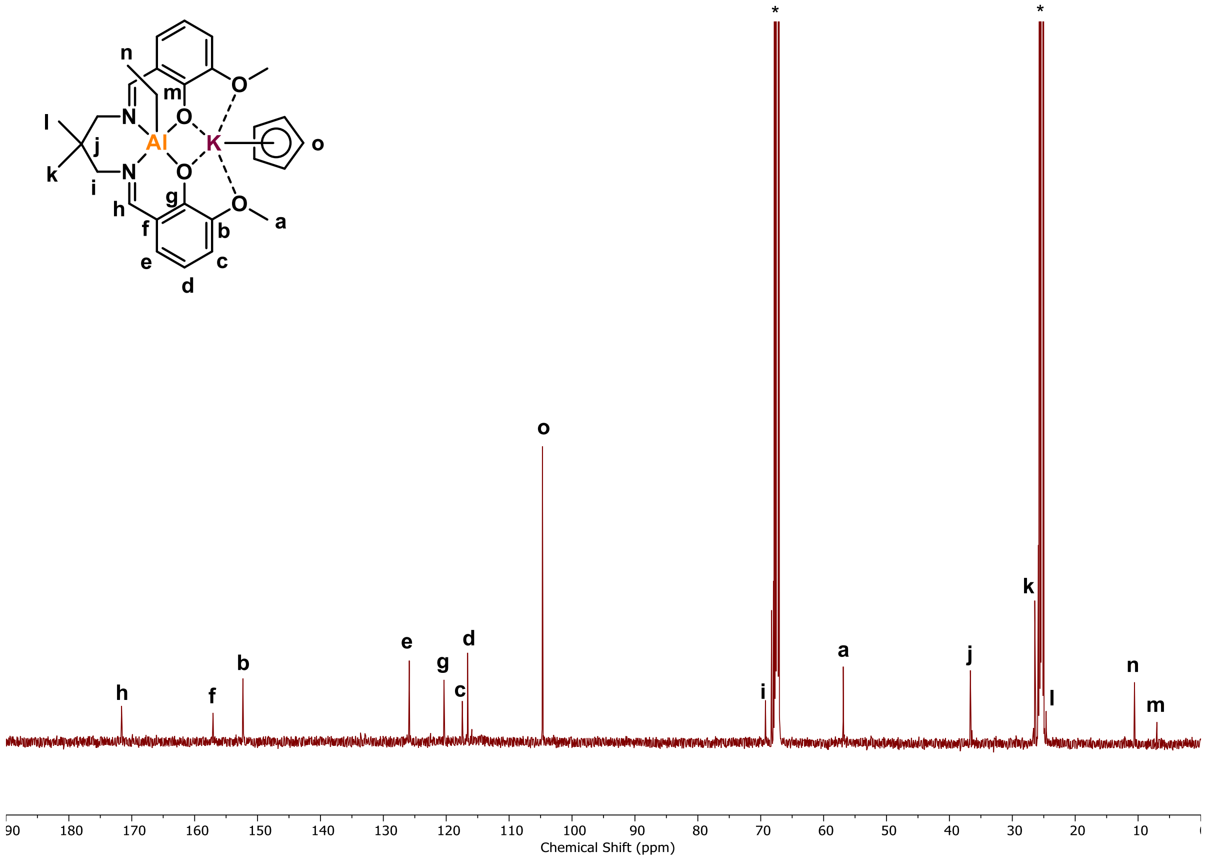

Figure S2. ^{13}C NMR spectrum (126 MHz, 298 K, THF-d8(*)) of complex **1**.

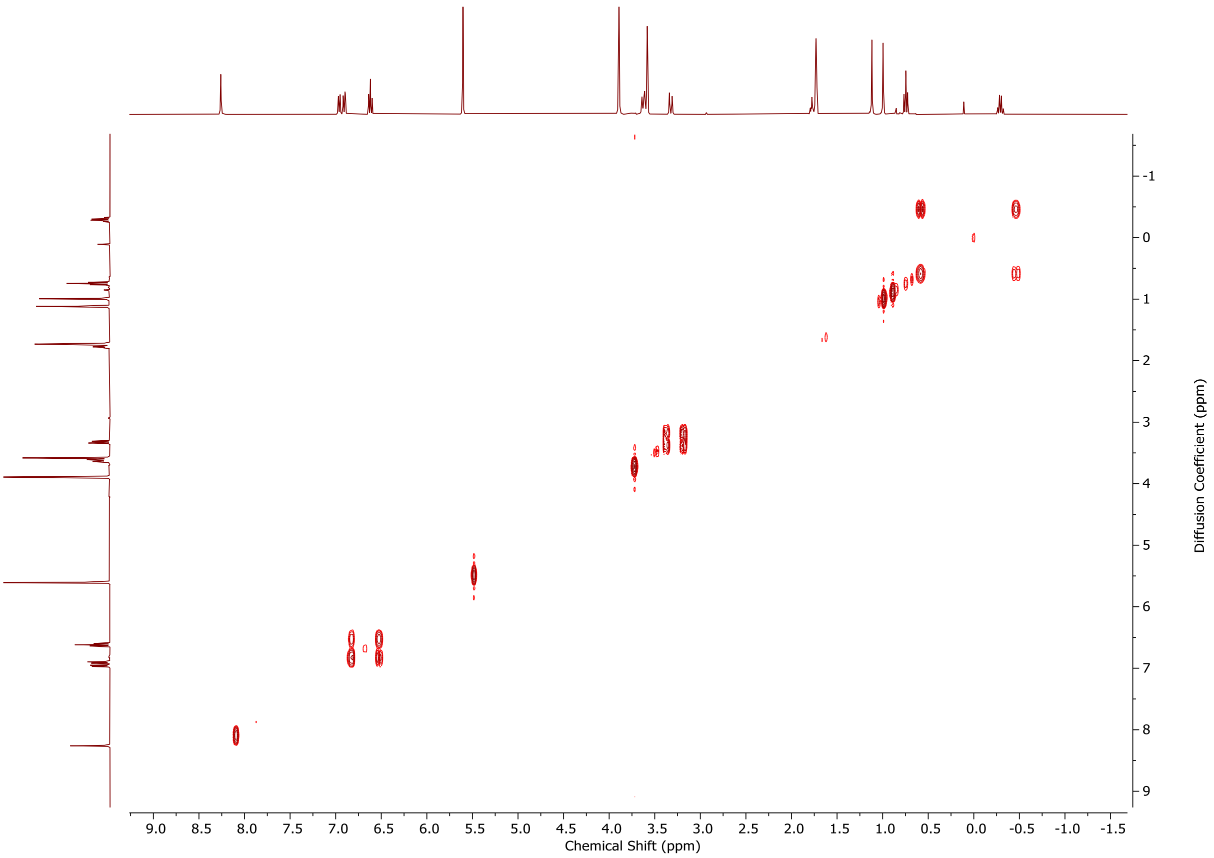

Figure S3. ^{1}H COSY NMR Spectrum (400 MHz, 298 K, THF-d_8) of complex **1**.

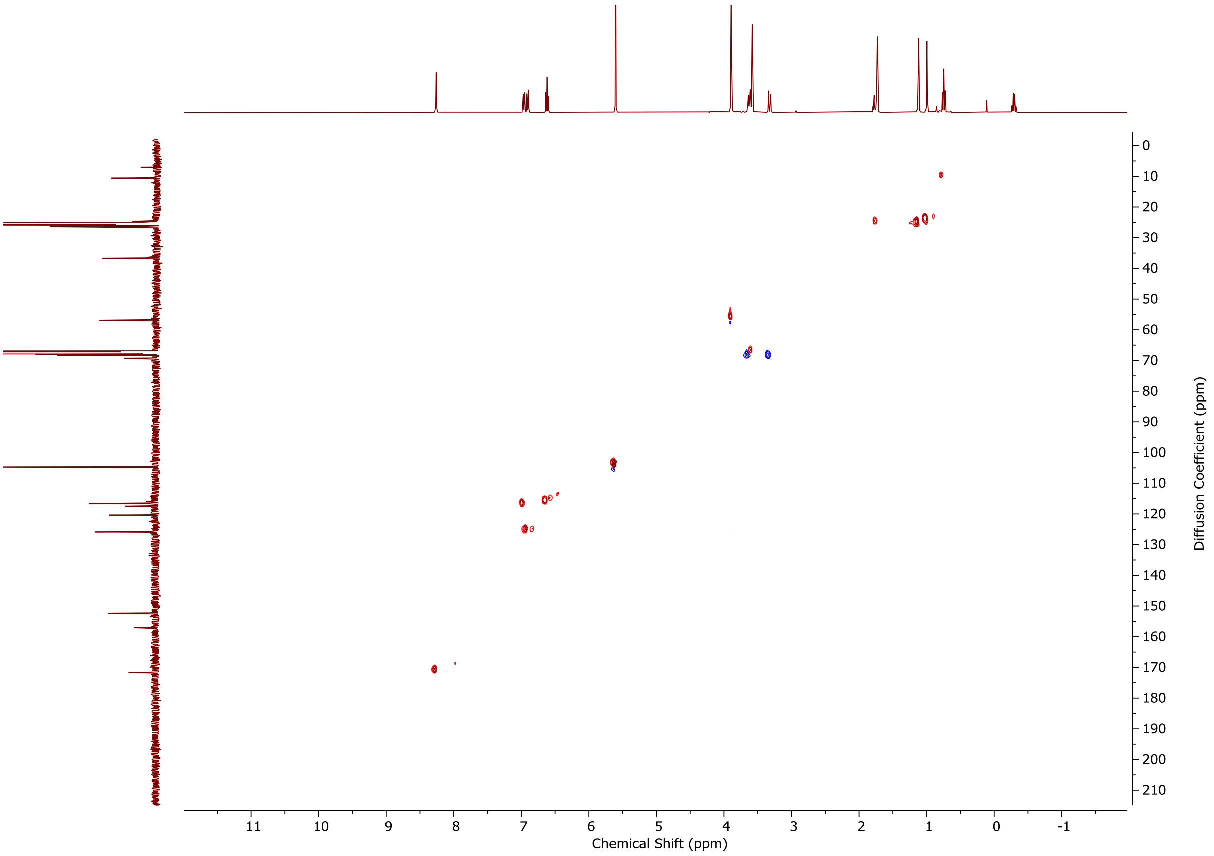

Figure S4. ^{1}H-^{13}C HSQC NMR Spectrum (298 K, THF-d_8) of complex **1**.

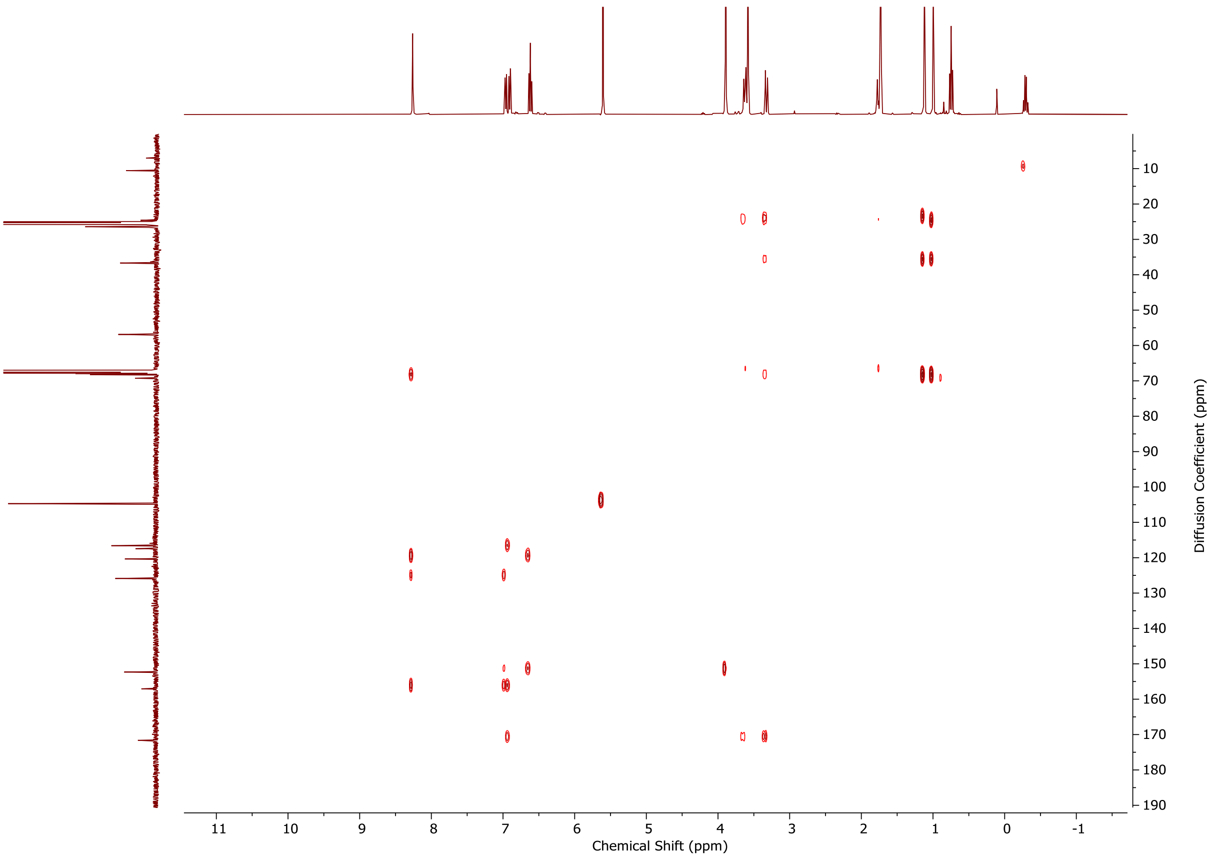

Figure S5. ^{1}H-^{13}C HMBC NMR Spectrum (298 K, THF-d_8) of complex **1**.

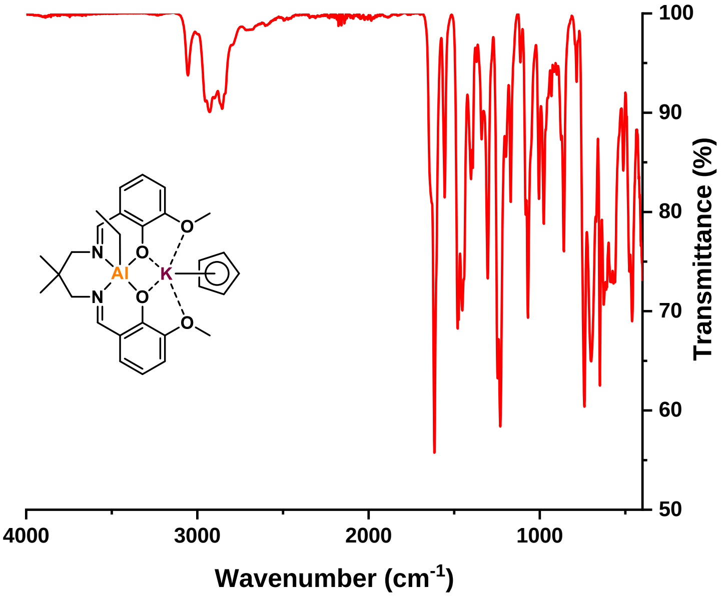

Figure S6. Infrared spectrum of complex **1**.

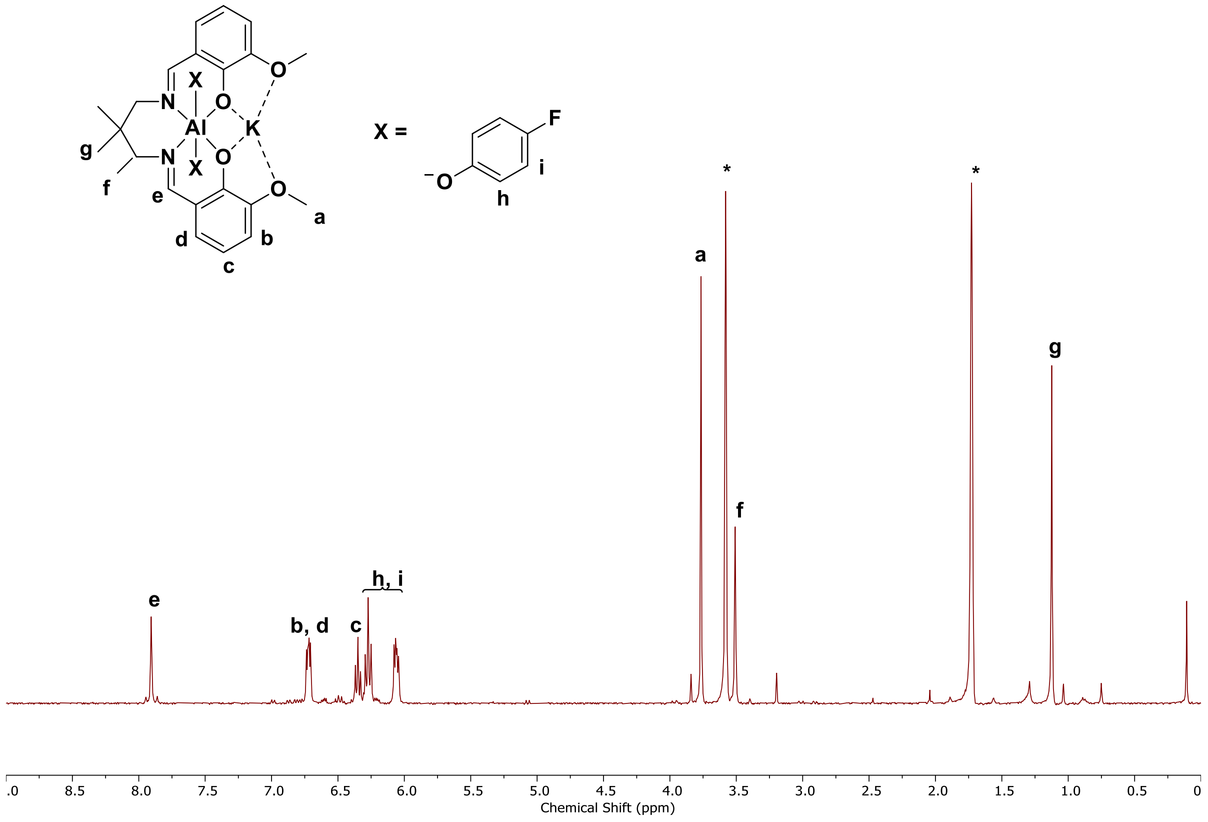

Figure S7. ^{1}H NMR spectrum (400 MHz, 298 K, THF-d_8) of complex **2**.

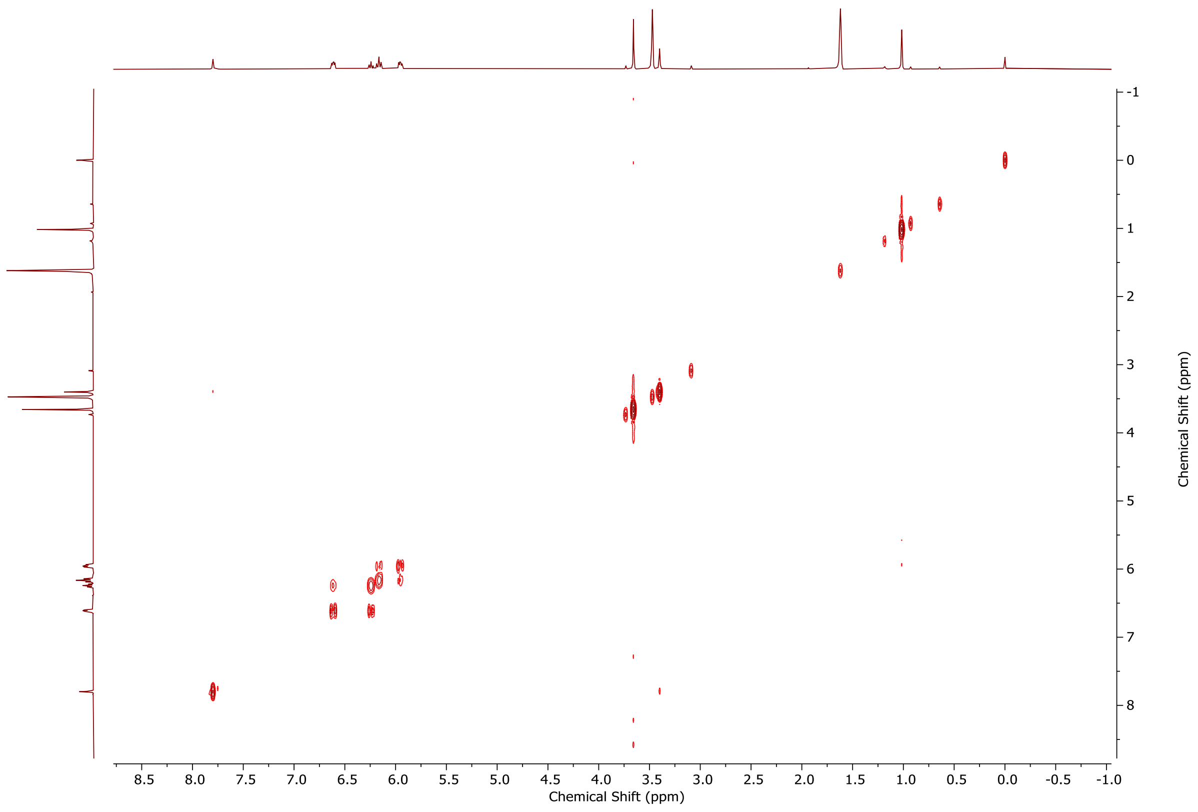

Figure S8. ^{1}H COSY NMR spectrum (400 MHz, 298 K, THF-d_8) of complex **2**.

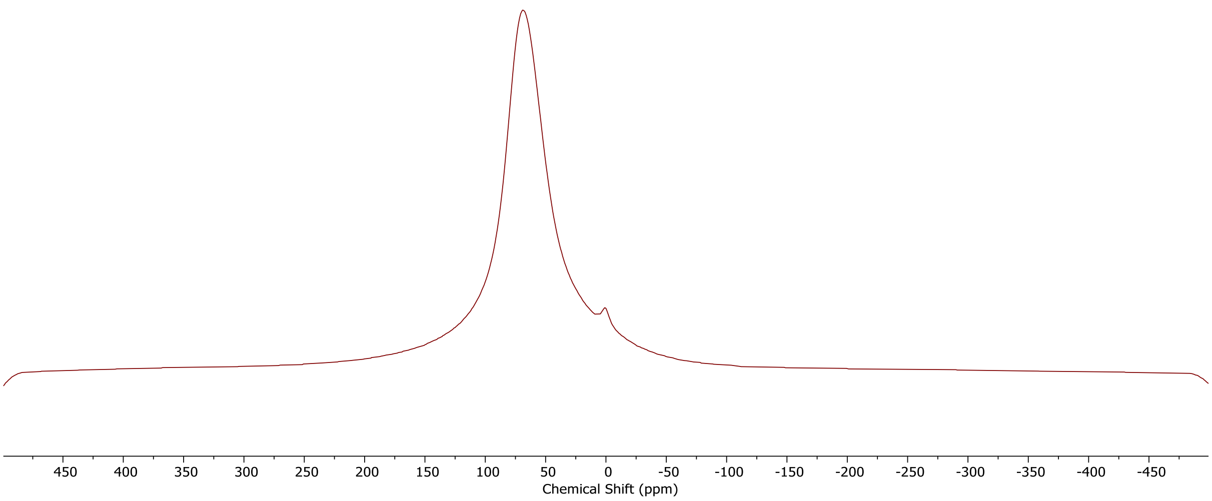

Figure S9. ^{27}Al NMR spectrum (104 MHz, 298 K, THF-d_8) of complex **2**.

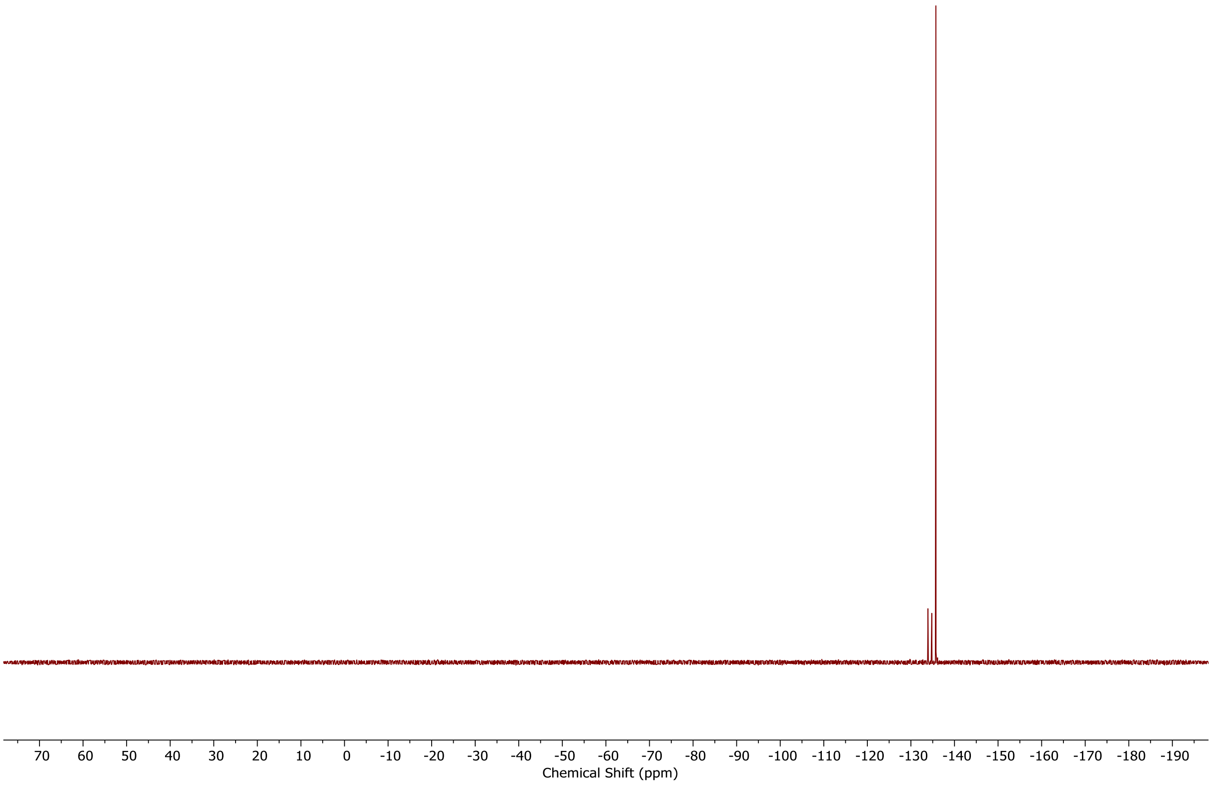

Figure S10. ^{19}F NMR spectrum (THF-d_8, 376 MHz, 298K) of complex **2**.

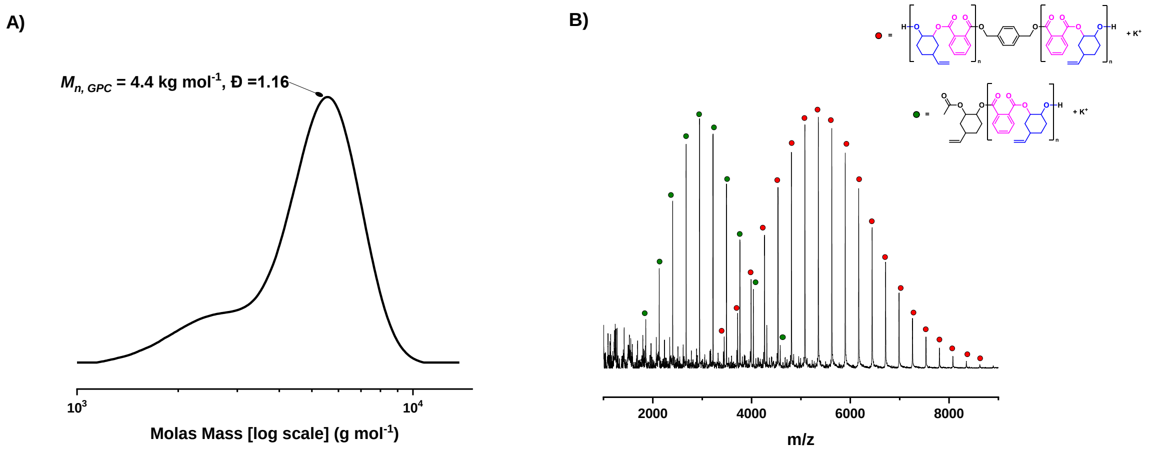

Figure S11. PvCHPE synthesised with [L_{van}AlK(OAc)$_2$] A) Molar mass distribution of PvCHPE obtained with [L_{van}AlK(OAc)$_2$]. Conditions: [L_{van}AlK(OAc)$_2$]:[BDM]:[PA]:[vCHO] = 1:4:100:2000, T = 100 °C. B) MALDI-ToF Spectrum of PvCHPE obtained. $M_{n,calc}$(repeat unit) = 272.3 g mol^{-1}, $M_{n,\ theoretical}$(repeat unit) = 272.3 g mol^{-1}; $M_{n,calc}$(end group) = 178.7 g mol^{-1}, $M_{n,\ theoretical}$(end group) = 177.3 g mol^{-1}.

Table S1. Aliquot data used to determine rates of epoxide/PA ROCOP with complex **1**.[a]

Entry	Epoxide	Temp (°C)	Time (m)	PA Conv.[b] (%)	TON[c]	TOF[d] (h^{-1})
1	CHO	100	10	43	172	1032
2	vCHO	100	45	99	396	528
3	tBGE	100	60	81	324	324
4	AGE	100	60	70	280	280
5	PO	60	360	26	104	17

[a] Conditions: [**1**]:[BDM]:[PA]:[Epoxide] = 1:4:400:2000. [b] Determined by ^{1}H NMR spectroscopy through comparison of resonances associated with PA (8.10 - 7.85 ppm) and associated polyester (typical range: 7.80 - 7.30 ppm).[4] [c] Turnover number (TON) = number of moles of anhydride consumed/number of moles of catalyst. [d] Turnover frequency (TOF) = TON/time (hours).

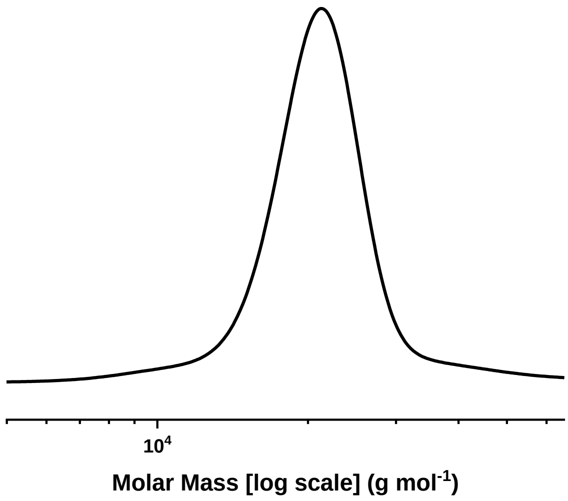

Figure S12. GPC trace of poly(CHO-*alt*-PA) obtained with complex **1**, corresponding to Table 1, Entry 1.

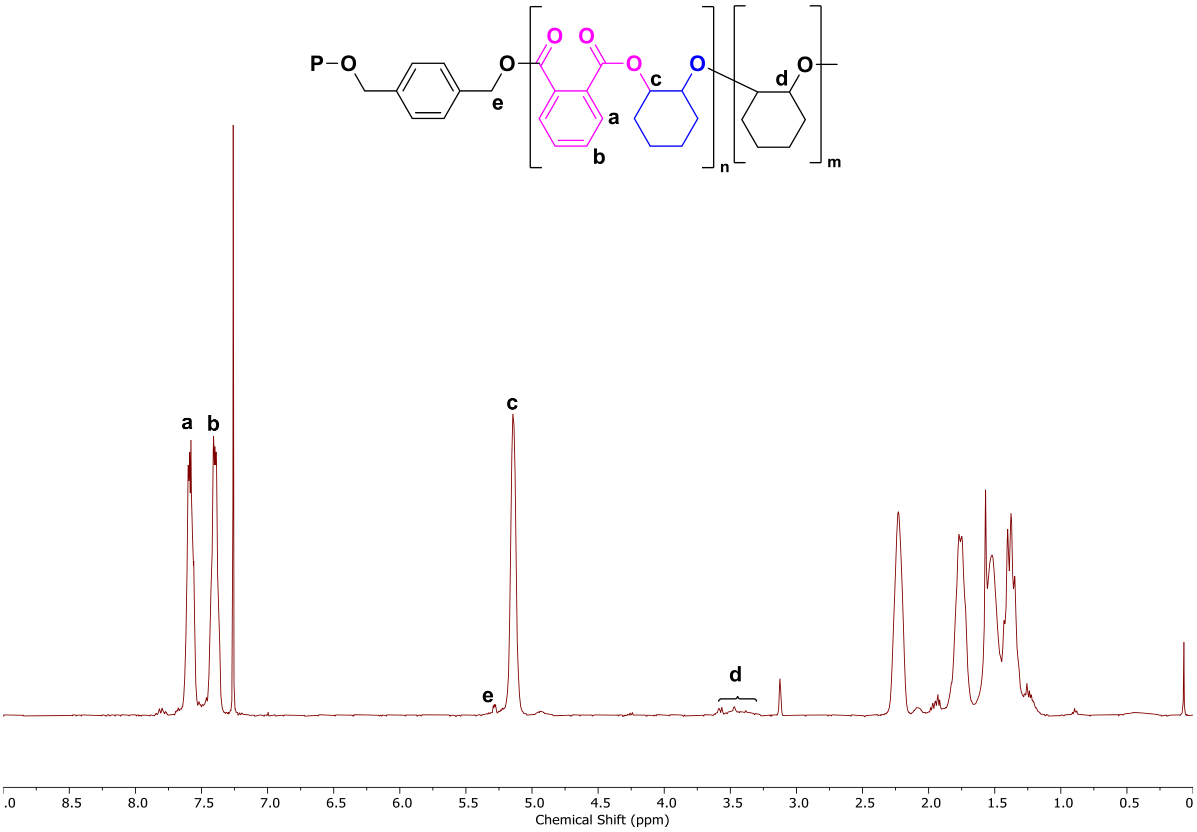

Figure S13. ^{1}H NMR spectrum ($CDCl_3$, 400 MHz, 298K) of isolated poly(CHO-*alt*-PA).

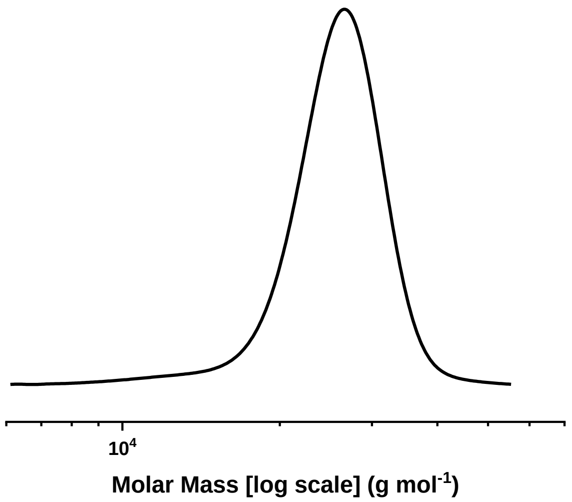

Figure S14. GPC trace of poly(vCHO-*alt*-PA) (PvCHPE) obtained with complex **1**, corresponding to Table 1, Entry 2.

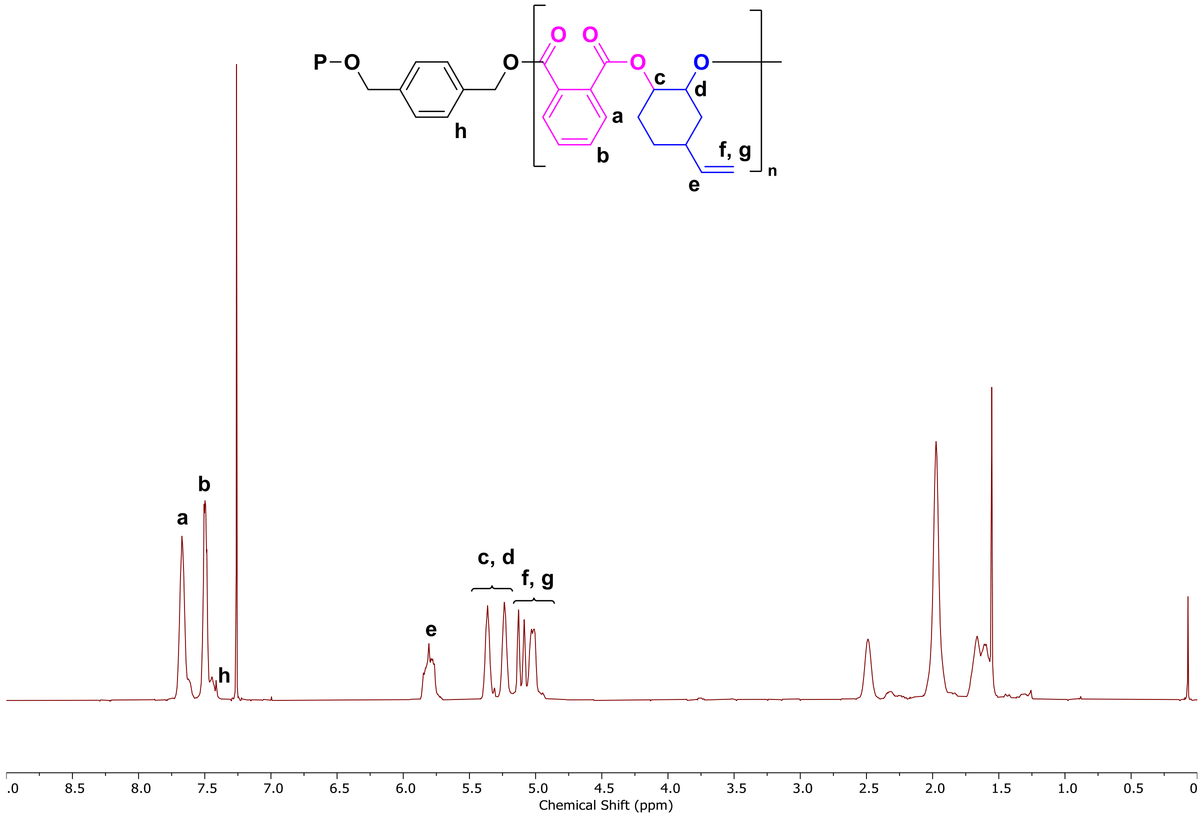

Figure S15. ^{1}H NMR spectrum ($CDCl_3$, 400 MHz, 298K) of isolated poly(vCHO-*alt*-PA).

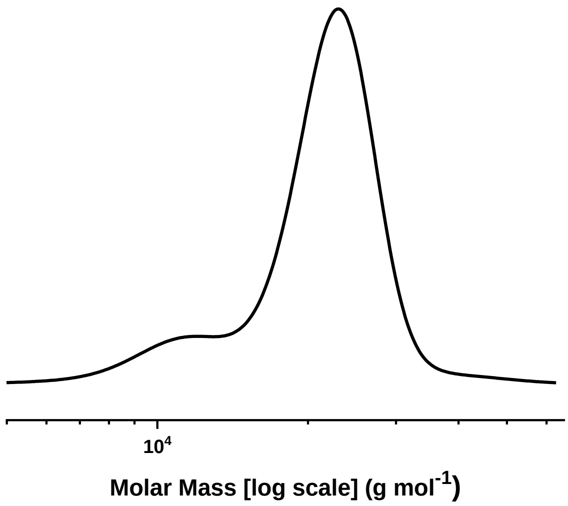

Figure S16. GPC trace of poly(tBGE-*alt*-PA) obtained with complex **1**, corresponding to Table 1, Entry 3. Low molar mass shouldering attributed to trace amounts of residual monofunctional alcohols.

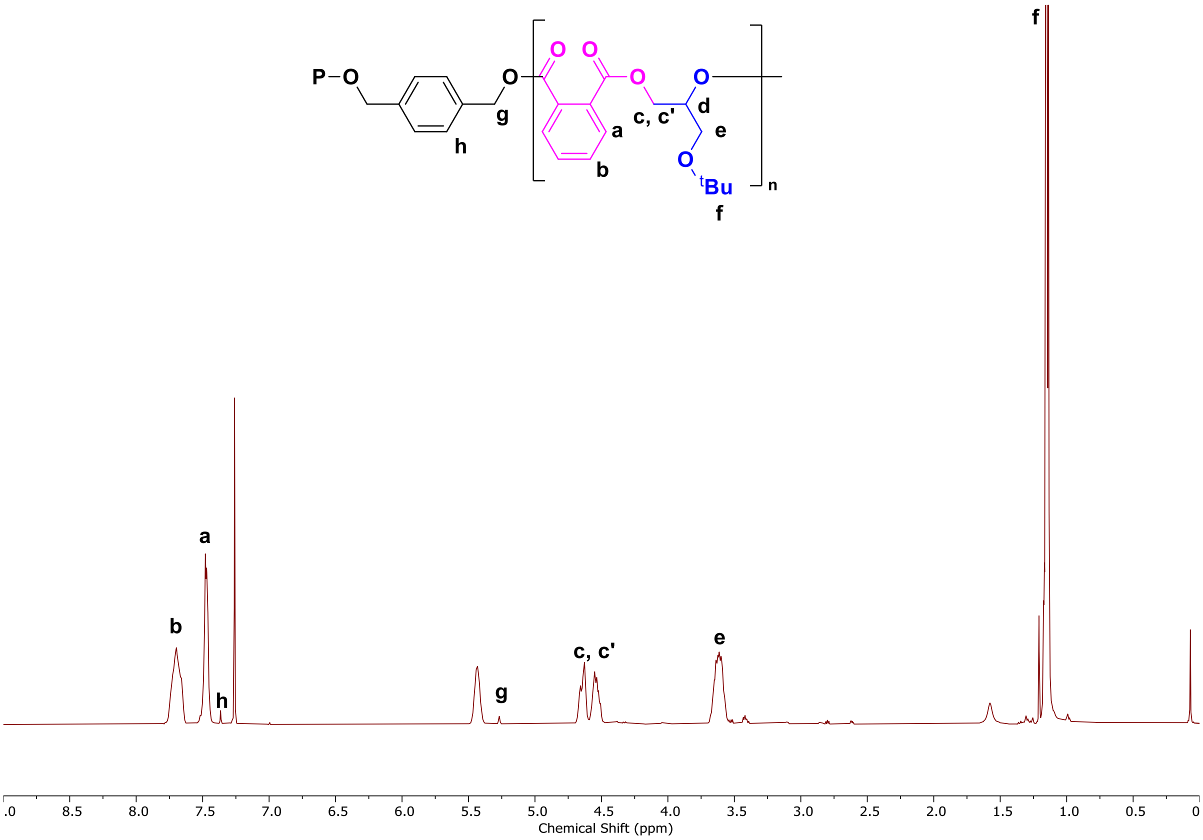

Figure S17. ^{1}H NMR spectrum ($CDCl_3$, 400 MHz, 298K) of isolated poly(tBGE-*alt*-PA).

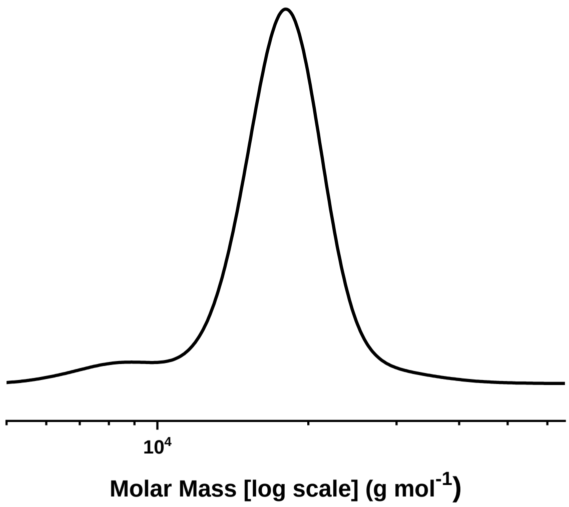

Figure S18. GPC trace of poly(AGE-*alt*-PA) obtained with complex **1**, corresponding to Table 1, Entry 4.

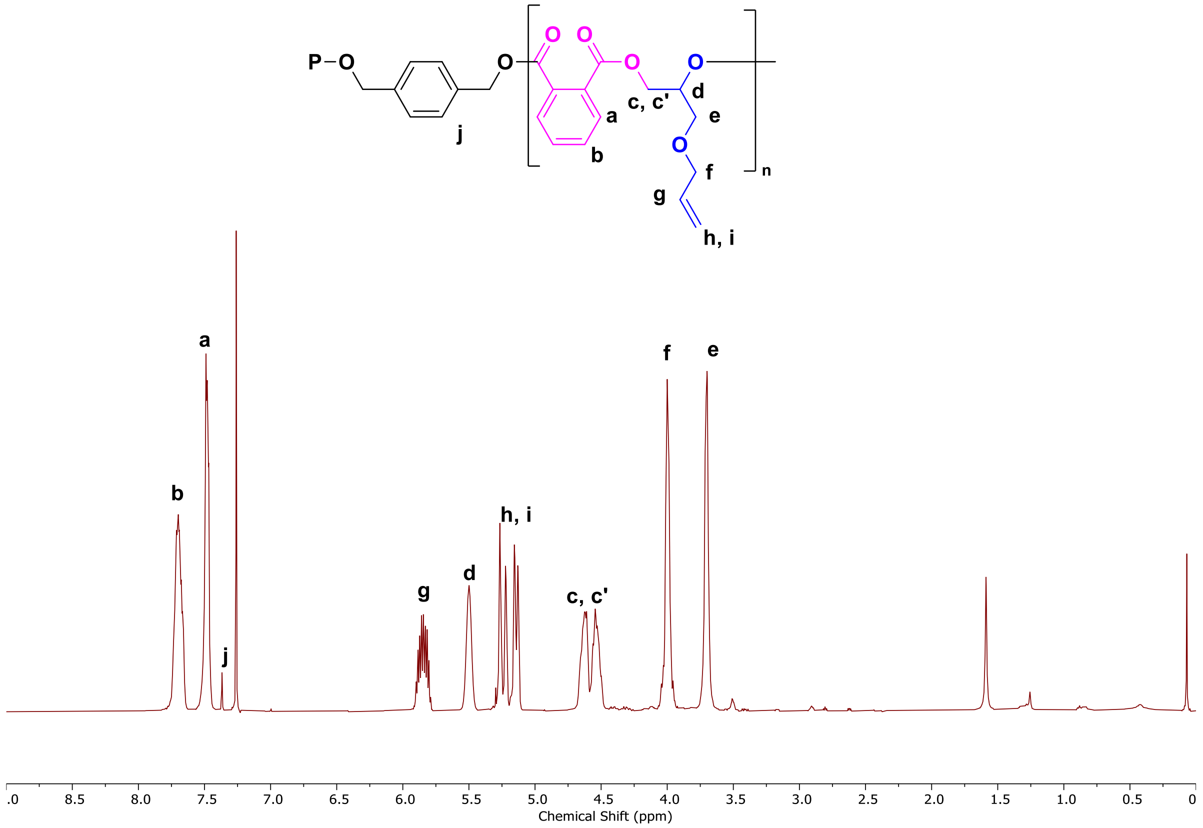

Figure S19. ^{1}H NMR spectrum ($CDCl_3$, 400 MHz, 298K) of isolated poly(AGE-*alt*-PA).

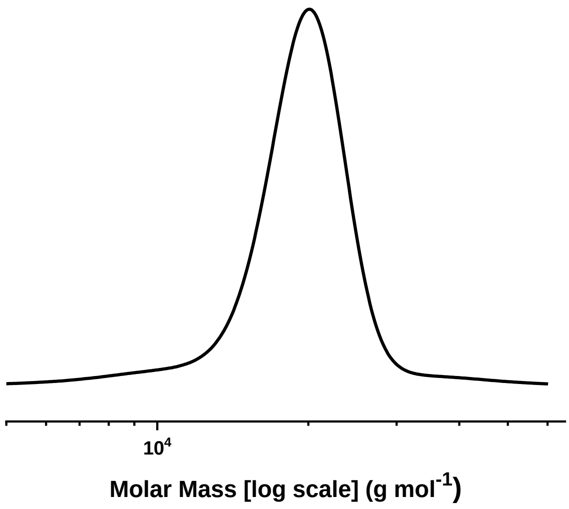

Figure S20. GPC trace of poly(PO-*alt*-PA) obtained with complex **1**, corresponding to Table 1, Entry 5.

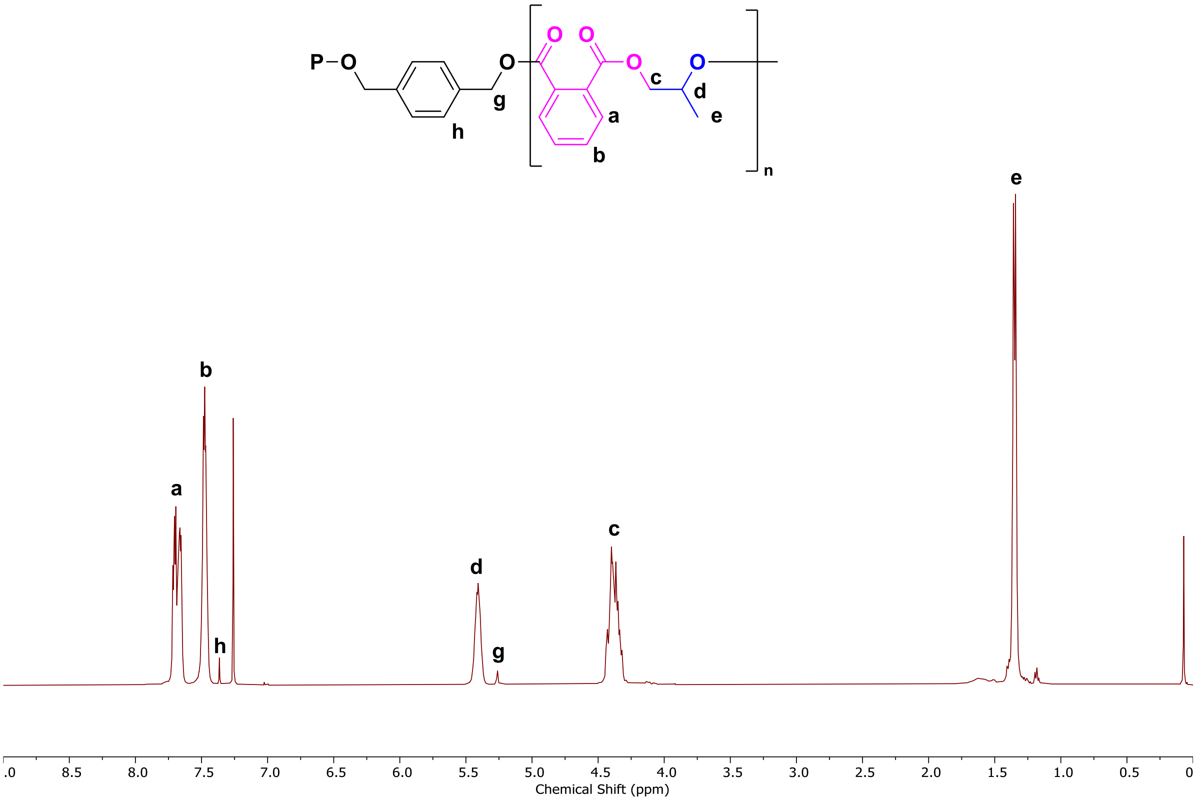

Figure S21. ^{1}H NMR spectrum ($CDCl_3$, 400 MHz, 298K) of isolated poly(PO-*alt*-PA).

Table S2. Synthesis of a triblock copolymer with complex **1**.[a]

Entry	Time (h)	Temp. (°C)	Conv. [PA, DL][b] (%)	$M_{n,\ GPC}$[c] (kg mol^{-1})	Đ[d]	$M_{n,Th}$[e]. (kg mol^{-1})
1	12	100	100, 92	80.8	1.04	95.7

[a] Conditions: [**1**]:[BDM]:[PA]:[vCHO]:[DL] = 1:4:400:2000:1600, T = 100 °C. [b] Determined by ^{1}H NMR spectroscopy through comparison of resonances associated with PA (8.10 - 7.85 ppm) and PvCHPE (7.75- 7.40 ppm) and ε-DL (4.27 – 4.17 ppm) and PDL (4.87 – 4.80 ppm). [c] Determined by GPC in tetrahydrofuran, at 30 °C, using narrow dispersity polystyrene standards. [d] Dispersity = M_w/M_n, determined by GPC in tetrahydrofuran, at 30 °C. [e] Theoretical molar mass, determined by [(TON$_{PA}$*$M_{n,\text{repeat unit,PvCHPE}}$ + TON$_{DL}$*$M_{n,\text{repeat unit,PDL}}$)/4]+$M_{n,BDM}$.

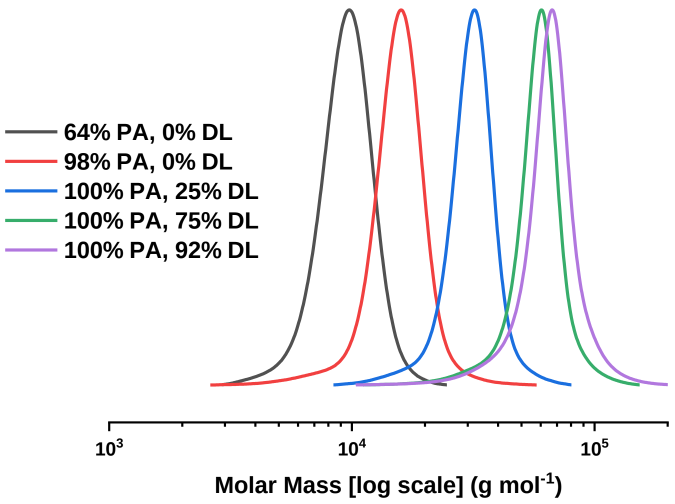

Figure S22. GPC Traces of aliquots taken during triblock synthesis, with monomer conversion determined by ^{1}H NMR of reaction aliquots, showing growth of a monodisperse triblock polyester.

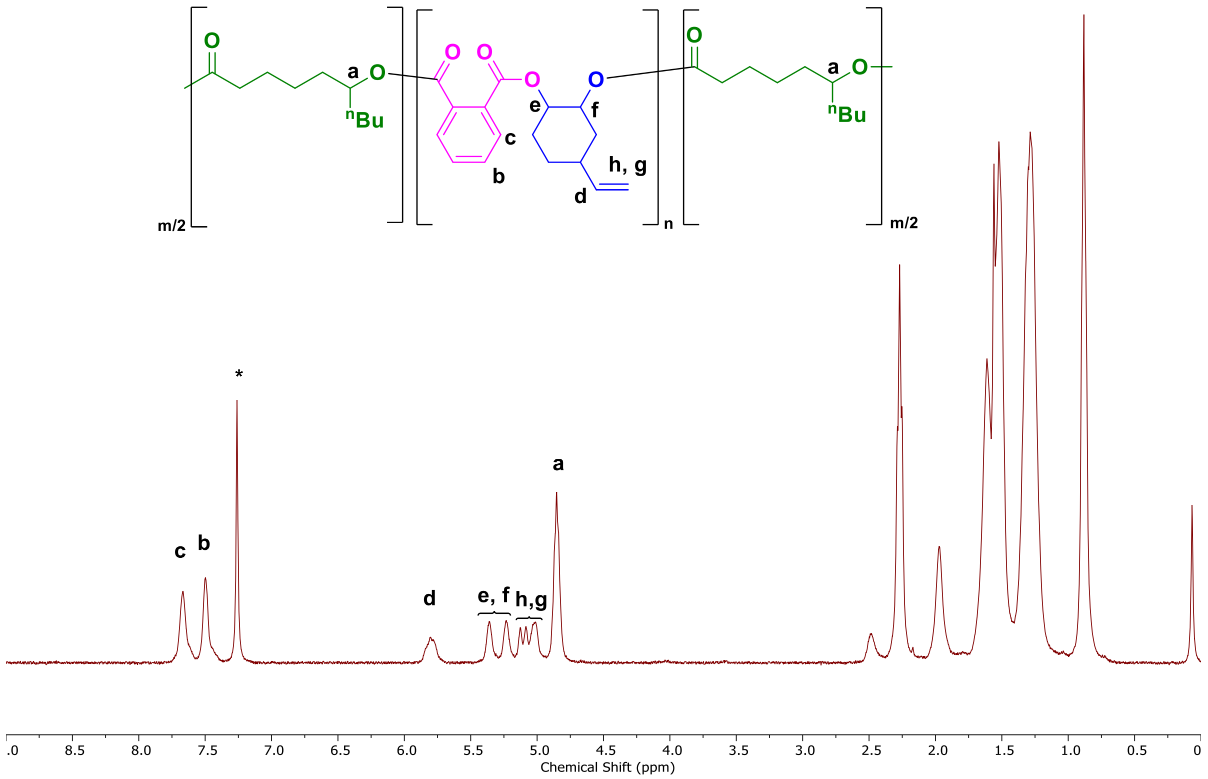

Figure S23. ^{1}H NMR spectrum ($CDCl_3$, 400 MHz, 298 K) of isolated PDL-*b*-PvCHPE-*b*-PDL synthesised with complex **1**.

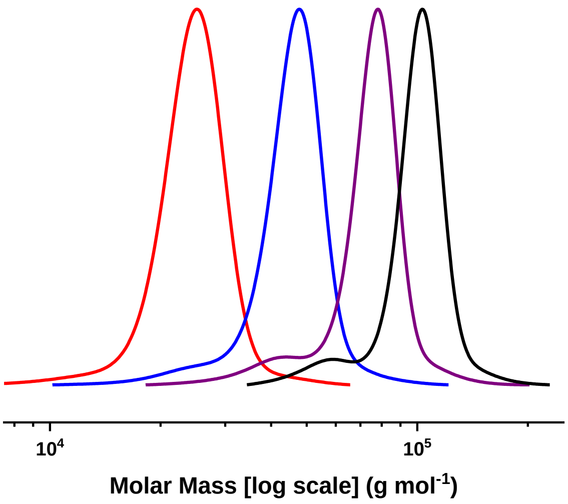

Figure S24. GPC Traces of PvCHPE-24, -44, -70 and -91, corresponding to Table 2, Entries 1 – 4, respectively. Minor low molar mass shouldering for the highest molar mass polymers is attributed to the presence of very low concentrations of monofunctional CTAs, such a vinyl cyclohexenol. Analogues of this have been previously observed in CHO, and are likely present in the chemical when purchased.[5, 6]

Taking the ratio of theoretical and experimental molecular mass:

$$\frac{M_{n,Th}}{M_{n,GPC}} = 1.25$$

Establish total amount of CTA equivs based on this ratio:

$$1.25 * [CTA]_{Added} = [CTA]_{Total} = 5$$

Back-calculate residual CTA equivalents:

$$[CTA]_{Total} = [CTA]_{Residual} + [CTA]_{Added}$$

$$[CTA]_{Residual} : [PA] : [vCHO] = 1 : 800 : 4000$$

Figure S25. Calculation of residual CTA, based on Table 2, Entry 2. This calculated ration is subsequently applied to Table 1, Entries 1, 3 and 4, showing a consistent amount of residual CTA in each polymerisation.

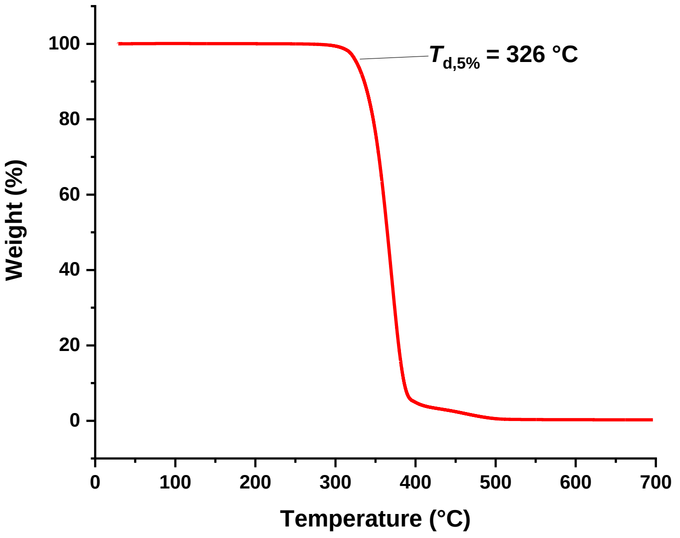

Figure S26. TGA Trace for PvCHPE-24.

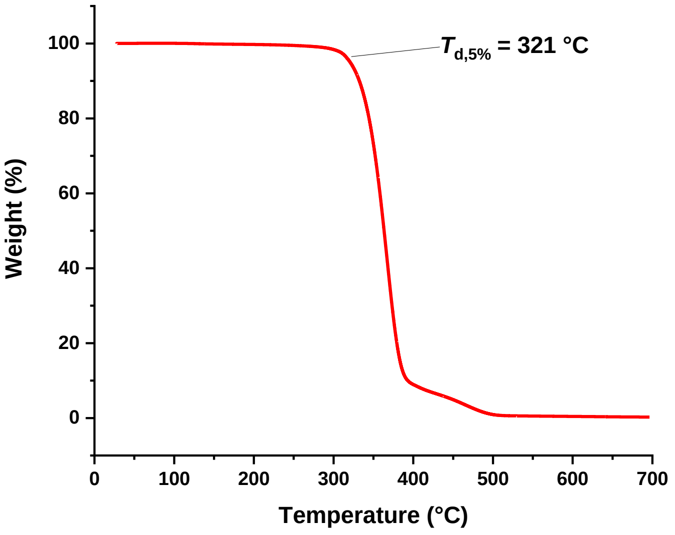

Figure S27. TGA Trace for PvCHPE-44.

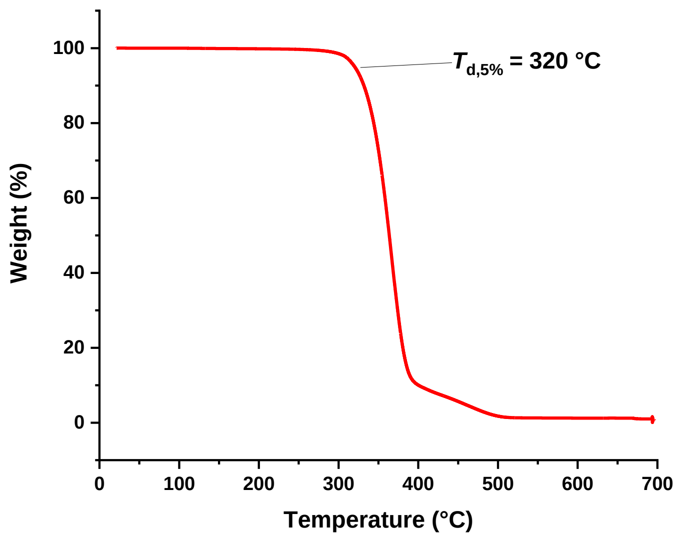

Figure S28. TGA Trace for PvCHPE-70.

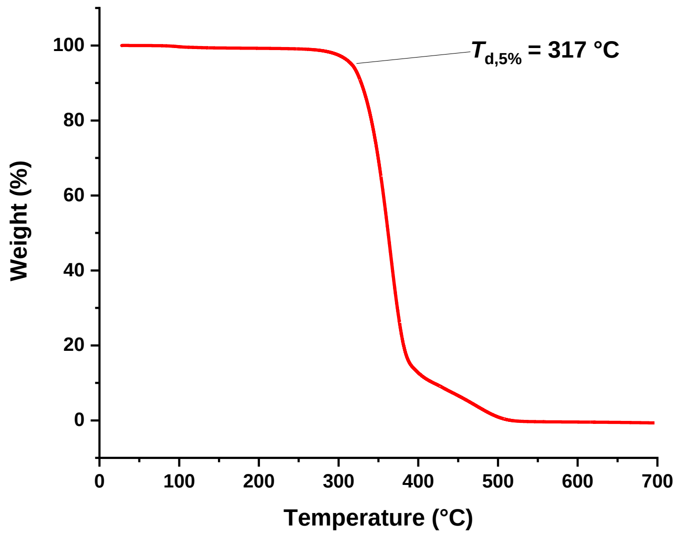

Figure S29. TGA Trace for PvCHPE-91.

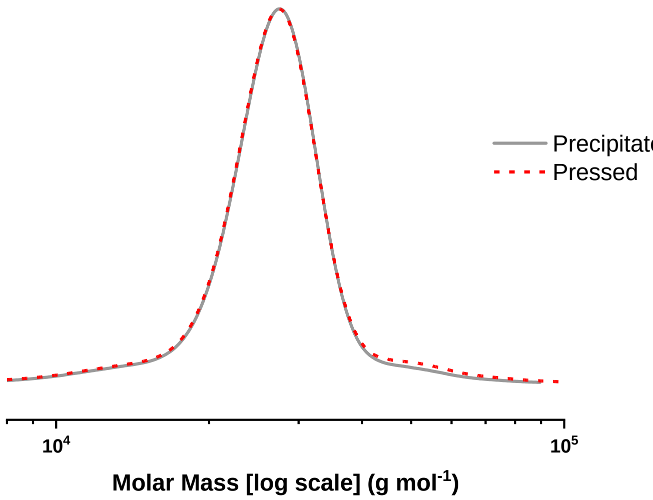

Figure S30. Typical GPC Trace of PvCHPE before and after processing *via* hot press (shown: PvCHPE-24).

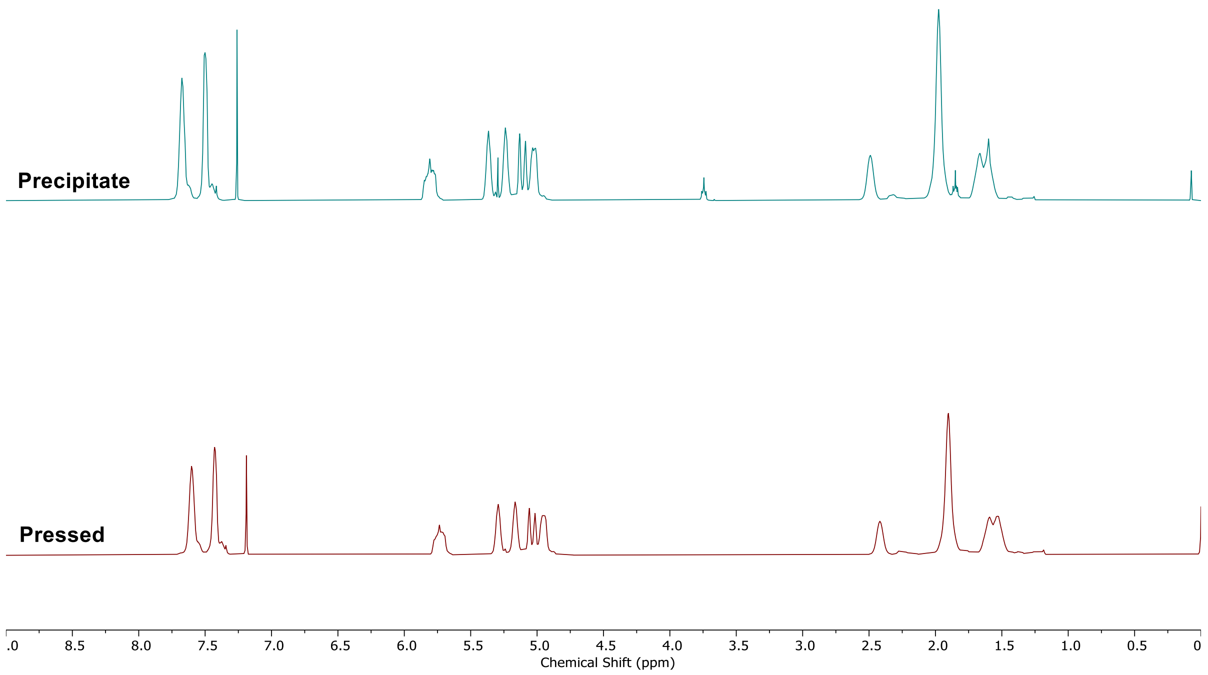

Figure S31. ^{1}H NMR ($CDCl_3$, 400 MHz, 298 K) of isolated PvCHPE-24 before and after processing *via* hot press.See Figure S15 for assignments.

Table S3. Mechanical Data for PvCHPE series analysed by Tension Film DMTA.

Entry	Name	$T_{g,\ DMTA}$[a] (°C)	E'[b] (GPa)	E''[c] (MPa)
1	PvCHPE-24	*N.d.*	*N.d.*	*N.d.*
2	PvCHPE-44	*N.d.*[d]	1.75 ± 0.05	59.1 ± 14.7
3	PvCHPE-70	152	2.04 ± 0.09	34.8 ± 9.1
4	PvCHPE-91	153	2.14 ± 0.08	24.3 ± 3.2

[a] Glass transition temperature measured by DMTA, taken from in tan(δ). [b] Tension storage modulus measured by DMTA (3 °C min^{-1}, 0.05% strain, 1 Hz) at 40 °C. [c] Tension loss modulus measured by DMTA (3 °C min^{-1}, 0.05% strain, 1 Hz) at 40 °C. [d]Samples yielded before T_g could be observed.

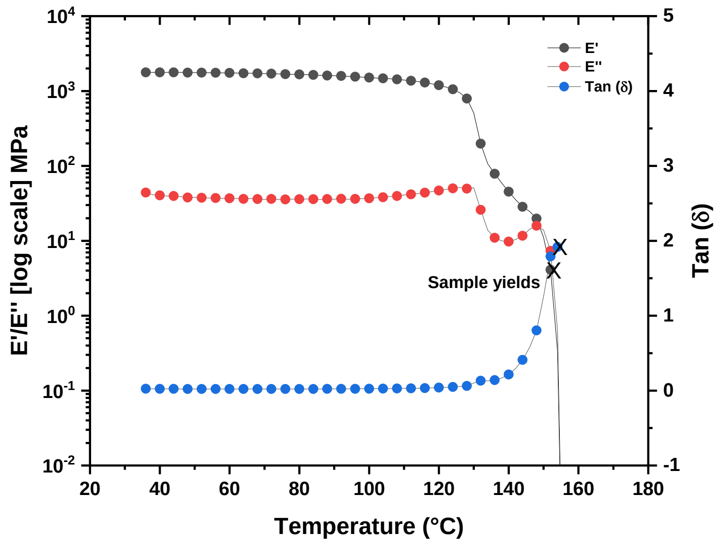

Figure S32. . Representative data from DMTA analysis of PvCHPE-44 *via* tension film methodology, between 30 – 200 °C. Storage Modulus (E'), Loss Modulus (E''), and Tan(δ) presented.

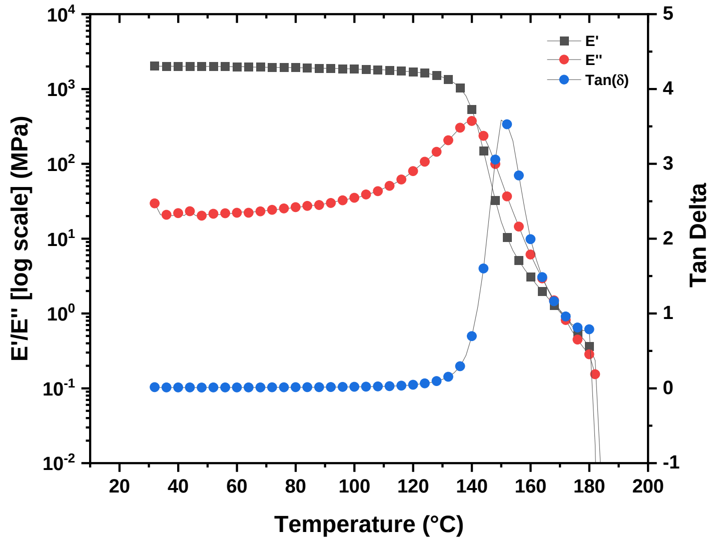

Figure S33. Representative data from DMTA analysis of PvCHPE-70 *via* tension film methodology, between 30 – 200 °C. Storage Modulus (E'), Loss Modulus (E''), and Tan(δ) presented.

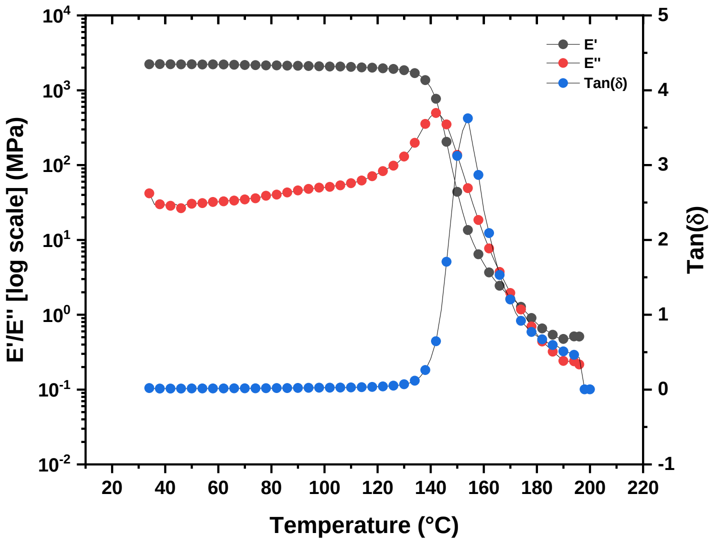

Figure S34. Representative data from DMTA analysis of PvCHPE-91 *via* tension film methodology, between 30 – 200 °C. Storage Modulus (E'), Loss Modulus (E''), and Tan(δ) presented.

References

1. T. K. Panda, M. T. Gamer and P. W. Roesky, *Organometallics*, 2003, **22**, 877-878.
2. A. Thevenon, J. A. Garden, A. J. P. White and C. K. Williams, *Inorg. Chem.*, 2015, **54**, 11906-11915.
3. W. T. Diment, T. Stosser, R. W. F. Kerr, A. Phanopoulos, C. B. Durr and C. K. Williams, *Catal. Sci. Technol.*, 2021, **11**, 1737-1745.
4. W. T. Diment, G. L. Gregory, R. W. F. Kerr, A. Phanopoulos, A. Buchard and C. K. Williams, *ACS Catal.*, 2021, **11**, 12532-12542.
5. J. Deng, M. Ratanasak, Y. Sako, H. Tokuda, C. Maeda, J. Y. Hasegawa, K. Nozaki and T. Ema, *Chem. Sci.*, 2020, **11**, 5669-5675.
6. H. Asaba, T. Iwasaki, M. Hatazawa, J. Deng, H. Nagae, K. Mashima and K. Nozaki, *Inorg. Chem.*, 2020, **59**, 7928-7933.
